# TOPAS, a network-based approach to detect disease modules in a top-down fashion

**DOI:** 10.1093/nargab/lqac093

**Published:** 2022-11-29

**Authors:** Davide Buzzao, Miguel Castresana-Aguirre, Dimitri Guala, Erik L L Sonnhammer

**Affiliations:** Department of Biochemistry and Biophysics, Stockholm University, Science for Life Laboratory, Box 1031, 171 21 Solna, Sweden; K7 Department of Oncology-Pathology, Karolinska Institute, 171 77 Stockholm, Sweden; Department of Biochemistry and Biophysics, Stockholm University, Science for Life Laboratory, Box 1031, 171 21 Solna, Sweden; Department of Biochemistry and Biophysics, Stockholm University, Science for Life Laboratory, Box 1031, 171 21 Solna, Sweden

## Abstract

A vast scenario of potential disease mechanisms and remedies is yet to be discovered. The field of Network Medicine has grown thanks to the massive amount of high-throughput data and the emerging evidence that disease-related proteins form ‘disease modules’. Relying on prior disease knowledge, network-based disease module detection algorithms aim at connecting the list of known disease associated genes by exploiting interaction networks. Most existing methods extend disease modules by iteratively adding connector genes in a bottom-up fashion, while top-down approaches remain largely unexplored. We have created TOPAS, an iterative approach that aims at connecting the largest number of seed nodes in a top-down fashion through connectors that guarantee the highest flow of a Random Walk with Restart in a network of functional associations. We used a corpus of 382 manually selected functional gene sets to benchmark our algorithm against SCA, DIAMOnD, MaxLink and ROBUST across four interactomes. We demonstrate that TOPAS outperforms competing methods in terms of Seed Recovery Rate, Seed to Connector Ratio and consistency during module detection. We also show that TOPAS achieves competitive performance in terms of biological relevance of detected modules and scalability.

## INTRODUCTION

Diseases have had a central role in society throughout history (1). More recently, the outbreak of the COVID-19 pandemic proved how lack of knowledge can heavily impact different aspects of the global community (2). For many common diseases, our understanding of the defective machinery that leads to the disease is still largely lacking. In the last decades, a massive amount of data has been generated using High-Throughput (HT) experimental techniques such as Next-Generation Sequencing (3) and Genome-Wide Association Studies (4), with the potential to uncover new disease mechanisms and their remedies. Unraveling these molecular disease mechanisms can be time- and resource-consuming if done with high-quality and small scale experiments. Moreover, there is mounting evidence that proteins, whenever related to the same diseases, participate together in the malfunctioning of the system they belong to (5–7). As a result, traditional techniques to research complex disorders, focusing on a particular protein or biological pathway are not cost-effective, nor can they, by definition, study the entire system. A more fruitful approach to understand the disease at the systems level could be computational modeling techniques.

Modeling biological systems using networks has been a successful technique to understand their structure and dynamics (8). Even a complex phenotype, e.g. a multigenic disease, can be represented as a network of interactions, the causative proteins being the vertices to link in the network. Indeed, disease-related genes have been shown to group together within a protein–protein interaction (PPI) network (5,6). A step further would be to use networks of functional associations that take into account the power of evidence integration and orthology information transfer between different species (9). It is from the application of network analysis methods to the study of human diseases that a new medical discipline, known as Network Medicine, stems (10). In this area of research, people have been focusing on expanding the knowledge about complex diseases by assuming that most disease gene sets are intrinsically incomplete. In this regard, a computational method that infers disease modules is said to be based on networks if it uses an interactome as input. Some of these methods use omics data derived from a disease to cluster the network into disease modules (11), while other methods build disease modules using a network and lists of known disease-associated genes, here referred to as ‘seed genes’. Such lists are usually curated by experts in the field and therefore represent a reliable foundation to base predictions on. Typically, a subnetwork of the seed genes and their interactions is first extracted, after which nodes are added according to their relevance in order to form a more complete disease module. Differentially expressed genes or co-expressed genes, as extracted from transcriptomic or methylomic HT experimental data, can also be used as an alternative to curated seed genes to explore complex diseases with network-based approaches at both the genetic and epigenetic levels (12).

SCA (13), DIAMOnD (14), MaxLink (15) and ROBUST (16) are all methods that can infer disease modules starting from a list of seed genes. Although DIAMOnD and SCA both construct their modules in an iterative bottom-up fashion, they are very different. DIAMOnD expands the seed set based on the significance of the added genes, whereas SCA aims at expanding the Largest Connected Component (LCC) of the seed set as much as possible with genes found among the direct neighbors of the seed genes. The limited search space of new potential disease genes allows SCA to minimize the number of putative connectors, also known as ‘seed connectors’. For both methods, each decision to add a connector is made on a local area of the network, which may be incomplete due to partial knowledge of the true underlying biological network, potentially leading to suboptimal selection of connectors. As decisions are made on only a fraction of nodes and links in the network, bottom-up methods are fast and use limited computational resources.

MaxLink is an approach to uncover new potential disease genes by examining the first order neighbors. It is studied here to get a baseline for the level of module detection that can be achieved from studying the immediate local network neighborhood of disease-associated genes. As with DIAMOnD, MaxLink often does not identify all seed genes as part of the final disease module, but rather discovers a large number of other potential disease genes. DOMINO (17), MuST (18) and ROBUST are top-down approaches that use Steiner trees for disease module detection. The objective of Steiner trees is to maximize the prize of collecting seed nodes in the disease module, but this does not necessarily find the maximum number of seed genes. Moreover, ROBUST runs by default in its most explorative configuration (16), which often results in the inclusion of a high number of seed connectors in the final module.

We here describe a novel network-based disease module detection algorithm that works in a top-down fashion. Our TOP-down Attachment of Seeds (TOPAS) algorithm combines the best parts of SCA and ROBUST by maximizing the number of seed genes while adding the fewest number of connectors in the final module. TOPAS uses the whole network as input and does preprocessing to ensure that the seed nodes are not a part of any disconnected subnetworks. Preprocessing scales approximately quadratically with the input size, but this is the only way to avoid the problems stemming from making decisions on a local area of the network. Despite the higher algorithmic complexity, the runtime of TOPAS is only seconds or minutes for almost all tested gene sets. Connectors are chosen in a later step ensuring that all seeds lie at a user-specified maximum allowable distance from each other, and that the selected connectors have the highest probability in Random Walk with Restart (RWR) runs in the global network. To fully exploit the potential of the interactome, connectors are iteratively removed from the whole network, rather than adding some to initially isolated seed genes. We benchmarked TOPAS against DIAMOnD, SCA, MaxLink and ROBUST on four different interactomes using a list of 70 complex diseases, a large corpus of targets of 220 drugs and a database of 92 disease-related pathways. Overall, TOPAS outperformed competing approaches both in terms of Seed Recovery Rate and Seed to Connector Ratio. We also demonstrate that TOPAS produces similar results in terms of scalability and biological relevance of detected modules. Furthermore, we assessed the similarity of the resulting modules across the different methods within the same network, as well as each method's consistency by using different underlying networks.

## MATERIALS AND METHODS

### Protein networks

#### The Ghiassian network

The Ghiassian network has been assembled by Ghiassian *et al.* (14) as an integration of eight different types of evidence of physical protein–protein (PPI) interactions. The used data sources include: protein–DNA regulatory interactions, literature-curated interactions, metabolic enzyme-coupled interactions, experimentally determined binary interactions and protein complexes, kinase-substrate pairs and signaling interactions. For each source of data an interactome was retrieved. The final network is the union of all eight interactomes that contains 13 460 proteins and 138 427 links, with a median node degree of 7.

#### FunCoup

The Functional Coupling (FunCoup) v5 is a resource of genome-wide functional association networks (19). It uses a unique redundancy-weighted naïve Bayesian integration to combine both direct and indirect interactions, exclusively supported by experimental data. It contrasts five gold standards against background interactions expected by chance to train Bayesian scoring functions for eleven different evidences of seven broad types (co-expression, co-localization, co-evolution, co-regulation, domain-domain interaction, genetic interaction, and PPI) and extract functional association networks for 21 species from all domains of life, extensively using transfer of evidence between species using Inparanoid (20) orthologs. Each link in the network comes with a confidence score. A default threshold of 0.8 was used to enrich the human network for high quality associations, which results in an interactome of 12,890 proteins and 612,276 associations, with a median node degree of 13.

#### HumanNet

HumanNet v3 is a functional network of human genes that includes inferred associations from experimental datasets of gene co-expression, PPIs, genetic interactions, protein domain co-occurrence and genomic context similarity (21). It employs curated annotations Inferred from Direct Assay (IDA) and from Mutant Phenotype (IMP) retrieved from Gene Ontology Biological Process (22) and pathway information using MetaCyc (23) to extract gold standard associations. Different combinations of evidence result in different interactomes: HumanNet-PI (PPI network) being the lowest layer of a three-tier hierarchical model, HumanNet-FC (functional gene network) the intermediate layer and HumanNet-XC (extended gene network by co-citation in PubMed articles) the final layer. We used HumanNet-XC because it is the most comprehensive model and showed best performance in an earlier evaluation (21). A log likelihood scoring scheme is used to assess each link in the network; we took 10% of the top links, resulting in 15 677 proteins and 112 549 associations with a median node degree of 9.

#### STRING

The Search Tool for the Retrieval of Interacting Genes/Proteins (STRING) v11.5 is the most popular resource of functional association networks, available for 14,094 species (24). Each interaction is the result of an integration of seven ‘evidence channels’, as well as orthology-based support via eggNOG (25). The evidences are related to computational association predictions (i.e. neighborhood, fusion and co-occurrence), functional genomics experiments or direct laboratory assays (i.e. co-expression and PPIs), curated knowledge (i.e. pathways and protein complexes) and text-mining (i.e. statistical co-occurrence analysis). A final ‘combined score’ is given to each link as the result of likelihoods of associations that are extracted from separated networks of single evidence. Each evidence is benchmarked on curated gold standard interactions from KEGG. The default ‘combined score’ threshold of 800 generated a human network of 14,221 proteins and 180,391 interactions, with a median node degree of 11.

#### Seed gene sets

A corpus of 382 manually curated functional gene sets was chosen to compare methods throughout the analysis. For any disease, drug and pathway under investigation, the corresponding genes are referred to as ‘seed genes’. The size of the Largest Connected Components (LCC) of gene sets was tested with a degree-aware subsampling test of 10 000 random samples. *P*-values were considered significant if lower than 0.05. To accommodate each network's gene vocabulary, *UP000005640_9606.idmapping* (https://www.uniprot.org/news/2021/02/10/release) and *9606.protein.aliases.v11.5.txt* (https://string-db.org/cgi/download?sessionId=bDexq6ZYgwXq&species_text=Homo+sapiens) were used to map *Gene ID* and *Gene Name* to *Ensembl* gene and protein identifiers, for FunCoup and STRING respectively. The size of the gene sets before and after mapping is summarized in Supplementary Tables S1–S4.

#### 70 diseases

The disease dataset was originally published by Ghiassian *et al.* (14). A collection of 70 human disorders with at least 20 disease associated genes was collected by integrating data from public sources of information such as Online Mendelian Inheritance in Man (OMIM) (26) and UniProtKB/SwissProt (27), together with the PheGenI GWAS catalog (28).

#### Drug targets

The dataset was generated by Loscalzo *et al.* (13). Drugs and drug targets were downloaded from DrugBank (29), Therapeutic Target Database (TTD) (30), and PharmGKB (*PharmGKB: The Pharmacogenetics and Pharmacogenomics Knowledge Base*, n.d.). Together with drug bioactivity information retrieved by sources such as ChEMBL (31), BindingDB (32), and the IUPHAR/BPS Guide to PHARMACOLOGY (GtoPdb) (33), a corpus of 220 drugs with at least 10 related targets was collected.

#### KEGG diseases

KEGG is a popular collection of 16 databases that contain genomic information, biological pathways, disease- and drug-related data. We used the R package KEGGREST (v.1.34.0) to retrieve 318 KEGG (Release 101.0+/02–20, February 22) human pathways with at least 15 associated genes, which corresponds to the size of 95% of all the pathways. Ninety two of these pathways were related to human diseases, and belonged to ten different subclasses (i.e. endocrine and metabolic disease; Neurodegenerative disease; Substance dependence; Infectious disease: bacterial; Infectious disease: parasitic; Infectious disease: viral; cancer: overview; cancer: specific types; immune disease; cardiovascular disease).

### Disease module finding methods

#### TOPAS

**Figure 1.**
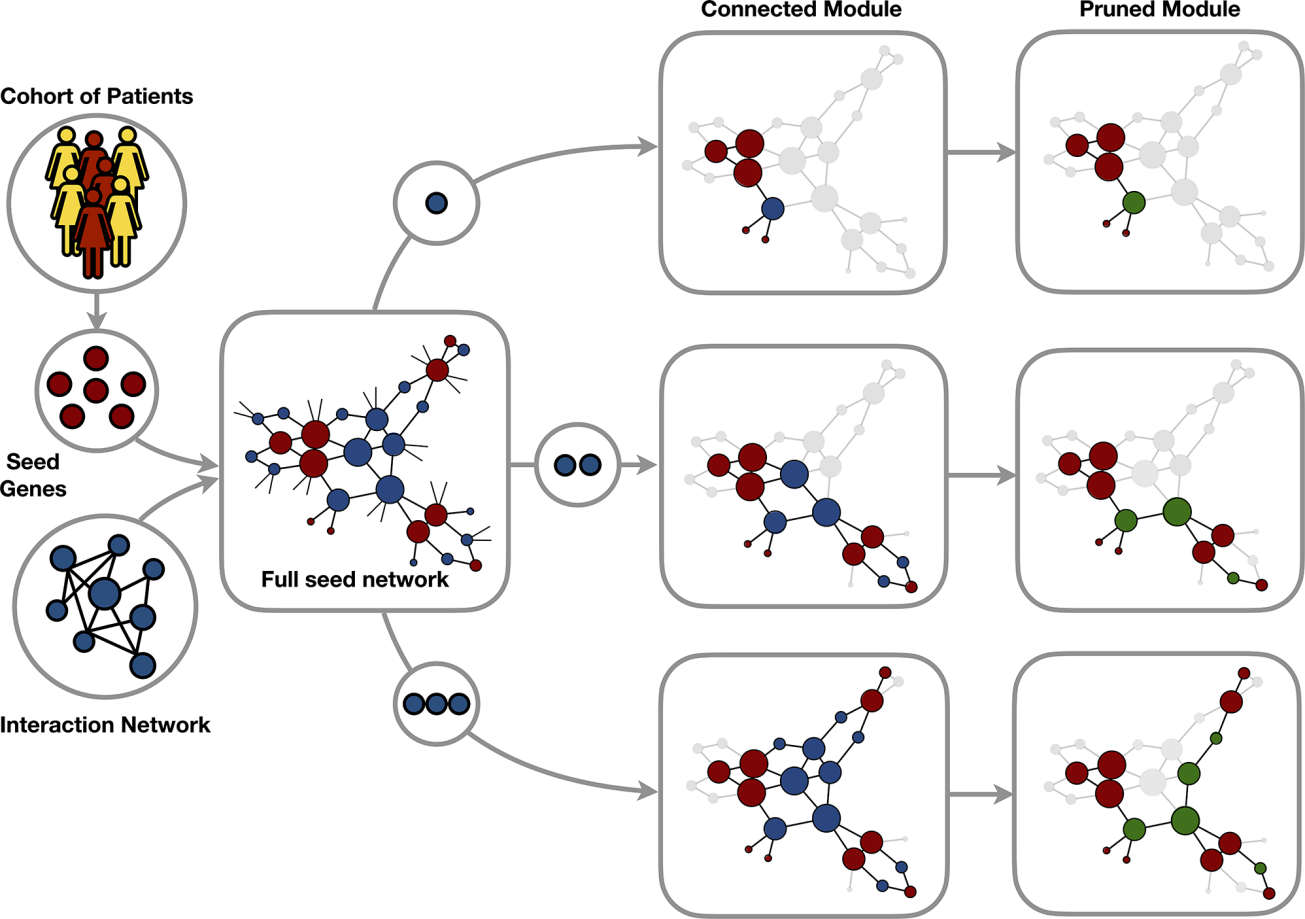
Illustration of the TOPAS workflow. The TOPAS disease module detection algorithm takes as input disease seed genes (in red) and an interaction network (in blue) to find disease-related connector genes (in green). TOPAS allows the user to input an integer parameter (e.g. 1, 2 or 3), that corresponds to the maximum number of allowed connector nodes between any two seeds, and pre-processes the network accordingly. It expands the seed coverage in the predicted module maximally and then prunes the connectors down to the minimal set that connects these seeds.

The TOP-down Attachment of Seeds (TOPAS) is an iterative approach that aims at connecting the largest number of seed nodes in a top-down fashion. The algorithm takes as input the whole network and preprocesses it to make sure seed nodes are not part of disconnected subnetworks. It considers a node to be a potential seed connector if it is found to be in a shortest path, of a user-specified maximum allowed distance, between any couple of seed nodes. To ignore functionally unrelated genes we suggest the maximum distance to be no larger than 3 (Figure 1). Selection of connectors happens in the step of network pruning, in which each node is initially assigned a probability value that increases with the flow through the node of a Random Walk with Restart (RWR) in the global network (34). Formally, the RWR is defined as in Equation (1),

(1) $$\begin{equation*}{p}^{t + 1} = ( {1 - r} ) A{p}^t + r{p}^0\end{equation*}$$

where the probability of being at node $i$ at time step $t$ is stored in the $i$-th element of the vector ${p}^t$, the initial probability vector ${p}^0$ being $1$s for seeds, $0$s otherwise. The row-normalized adjacency matrix $A$ will dictate the flow of the random walk, that in the RWR paradigm can restart by going to any randomly chosen seed node in the graph with a predetermined restart probability $r$ (by default set to 0.75). The resulting probability vector $p$ will at steady state contain the probability of RWR ending in each network node, that can be used to extract a global network-distance measure in a similar way as Köhler et al. and prioritize genes according to their location relative to known disease genes (35). The probabilities to end up at a certain node at the steady-state are here used to rank the pool of connectors. If we assume that the set of seed nodes $S = \{ {{s}_1,{s}_2, \ldots ,{s}_N} \}$ together with the ranked pool of potential connectors $P = \{ {{p}_1,{p}_2, \ldots ,{p}_M} \}$ induce a subnetwork ${G}_s$, the algorithm is an iterative process that scales approximately quadratically with the number of seeds and can be described as follows:

- temporarily delete ${p}_i$ from ${G}_s$ and extract the connected components of ${G}_s$;
- update ${G}_s$ by removing ${p}_i$, and its links, if the number of connected components remains unaltered.

Steps I-II are repeated $M$ times so that all putative connectors are tested. The final subnetwork is the predicted disease module. TOPAS is implemented in the R language (v4.1.0): the graph handling, the computation of the shortest paths and the connected components are done with the R package igraph (v.1.2.11), and the RWR is run using the R package dnet (v.1.1.7). TOPAS allows for parallelization during shortest path calculation using the R packages dosnow (v.1.0.20) and foreach (v.1.5.1).

#### Other disease module finding methods

We selected four methods that do not rely on any other information other than seed genes and a network to infer a disease module. SCA, DIAMOnD and MaxLink are bottom-up approaches. SCA aims at greedily connecting as many seed genes as possible, minimizing the number of connectors. DIAMOnD and MaxLink extend the seeds based on the significance of connectors. ROBUST is a top-down approach that enumerates diverse prize-collecting Steiner trees to robustify module detection.

#### DIAMOnD

The goal of the DIseAse MOdule Detection algorithm (DIAMOnD) is to determine which nodes in the network are more significantly related to the seed nodes. For a preset number of times (here 200, as suggested by the authors) DIAMOnD evaluates the significance of connection of all the first seeds’ neighbors with a hypergeometric statistical test to expand the module, one node at a time. At each iteration the proteins are ordered according to their *P*-values, these being computed as the probability of each single node to share more links to seed nodes than expected by chance; the one with the highest rank (i.e. lowest *P*-value) is then added to set of seed nodes. Also, the hyperparameter *alpha* can be chosen to downweight the selection of high degree nodes, here *alpha* is set to the default null contribution ($\alpha \ = \ 1$). The output of DIAMOnD is the ranked list of all the prioritized nodes, not necessarily belonging to a single predicted submodule. In the following analysis, only the largest predicted module is taken into account. The algorithm has been shown to retrieve fairly well connected disease modules, however with low coverage of seed nodes and a low seed to connector node ratio, decreasing with the allowed size of the predicted module. DIAMOnD (http://diamond.barabasilab.com) was run in a Python (v.3.9.10) version-controlled conda environment with networkx (v.2.6.3), numpy (v.1.22.1) and scipy (v.1.7.3).

#### MaxLink

Similarly to DIAMOnD, the Maxlink algorithm aims at identifying candidate genes that are enriched in direct connections to the set of seed genes. MaxLink considers all first neighbors of seed genes to be candidate gene connectors and all candidates are given a score based on the number of links to the seeds. The algorithm then uses a hypergeometric test to assess which candidates have a statistically significant set of interactions with the seeds. The genes having a *P*-value of less than 0.05 are retained. Only the largest predicted module is taken into account in the following analysis. A local implementation of MaxLink was run in a Python (v.3.9.10) version-controlled conda environment with networkx (v.2.6.3), pandas (v.1.4.0) and scipy (v.1.7.3).

#### ROBUST

The ROBUST disease module detection algorithm is designed to find a robust network of seeds via enumeration of $n \in \mathbb{N}$ prize-collecting Steiner trees (PCST), each tree being a near-optimal approximation of the PCST problem. After having collected trees, the final module consists of the set of genes found in $\tau \in ( {0,1} ]$ trees. The method has two other parameters: $\alpha \in ( {0,1} ]$ and $\beta \in [ {0,1} )$, which regulate the connector integration ratio and modify the decrease of the value for integrating the connector genes in the tree, respectively. Here ROBUST was run with default parameters, $\alpha ,\beta ,\ n,\ \tau = 0.25,\ 0.9,\ 30,\ 0.1$. In general, this approach yields disease modules with a high coverage of seeds and a lower Seed to Connector Ratio. ROBUST (https://github.com/bionetslab/robust) was run in a Python (v.3.7) version-controlled conda environment according to the author instructions.

#### SCA

The Seed Connector Algorithm (SCA) has the goal to link as many seed nodes as feasible using the fewest possible number of seed connectors. The pool of seed connectors is preemptively specified as the collection of all first neighbors of the seeds, and does not change throughout the algorithm. As an iterative, bottom-up approach the final module is retrieved by adding the seed connectors that increase the size of the LCC of the growing subnetwork starting with the seed subnetwork, one node at a time. The output of SCA is the list of all the seed and the prioritized seed connector nodes, not necessarily belonging to a single predicted submodule. This approach yields a disease module with a high coverage of seeds and a high seed protein to connector protein ratio. SCA is adapted to run in a Python (v.3.9.10) version-controlled conda environment with networkx (v.2.6.3).

### The benchmark

#### Scoring metrics

The Seed Recovery Rate (SRR) is computed as the ratio of recovered seed genes in the corresponding largest predicted module and the total number of seeds in the same network connected component (i.e. *connectable seeds*). The Seed to Connector Ratio (SCR) is calculated as the ratio of seed and seed connector nodes in the largest predicted seed module.

#### Biological relevance of predicted disease modules

We used the R package KEGGREST (v.1.34.0) to retrieve 318 KEGG (Release 101.0+/02–20, February 22) human pathways with at least 15 associated genes, which is 95% of all pathways. We mapped the retrieved pathway terms on the genes of the predicted modules. We used Infomap (36) in its default configuration, implemented in igraph v.1.2.11, to separate modules into more closely related genes. Infomap is a popular network-based clustering method that determines the ideal modular structure of the network by describing a random walk in the network with the minimum amount of necessary information. A degree-aware subsampling test of 10,000 random samples is used to assess the significance of the observed overlap of pathway terms between seeds and connectors. The overlaps are expressed as Jaccard index (Eq. 2) and only Benjamini-Hochberg FDR-corrected *P*-values of <0.05 were retained.

(2) $$\begin{equation*}J( {{\rm{A}},{\rm{B}}} ) = \frac{{\left| {{\rm{A}} \cap {\rm{B}}} \right|}}{{\left| {A \cup B} \right|}}\end{equation*}$$

#### Consistency between methods and between networks

We compared the benchmarked methods in terms of inference similarity by looking at the overlap of connector genes in the final predicted modules. In a similar way, we wanted to check method consistency with respect to module predictions. We therefore measured the overlap of genes in the inferred modules between combinations of underlying networks in use. The overlap was evaluated using the Jaccard index (Eq. 2) and the Szymkiewicz–Simpson coefficient (Eq. 3).

(3) $$\begin{equation*}overlap( {{\rm{A}},{\rm{B}}} ) = \frac{{\left| {{\rm{A}} \cap {\rm{B}}} \right|}}{{{\rm{min}}\left( {\left| A \right|,\left| B \right|} \right)}}\ \end{equation*}$$

#### Runtime

We ran TOPAS, SCA, ROBUST, DIAMOnD, and MaxLink in a macOS Monterey (v.12.2.1) system with a 2.7 GHz Quad-Core Intel Core i7 processor (16GB RAM). We extracted runtime by using the shell program *time* for each module detected and showed CPU-user and real time in seconds versus size of mapped seeds in the network.

## RESULTS

We present a novel algorithm TOPAS for connecting disease genes into a network module, and compare its performance to four other approaches. Each method takes as input a set of seed genes for each disease as well as a protein network. For the evaluation we used in total 382 gene sets of three types, and four networks. We will refer to three different configurations of TOPAS as TOPAS(1), TOPAS(2) and TOPAS(3) that respectively allow for one, two, or three potential connector(s) in a shortest path between any two of seeds.

### Network and disease gene set properties

**Figure 2.**
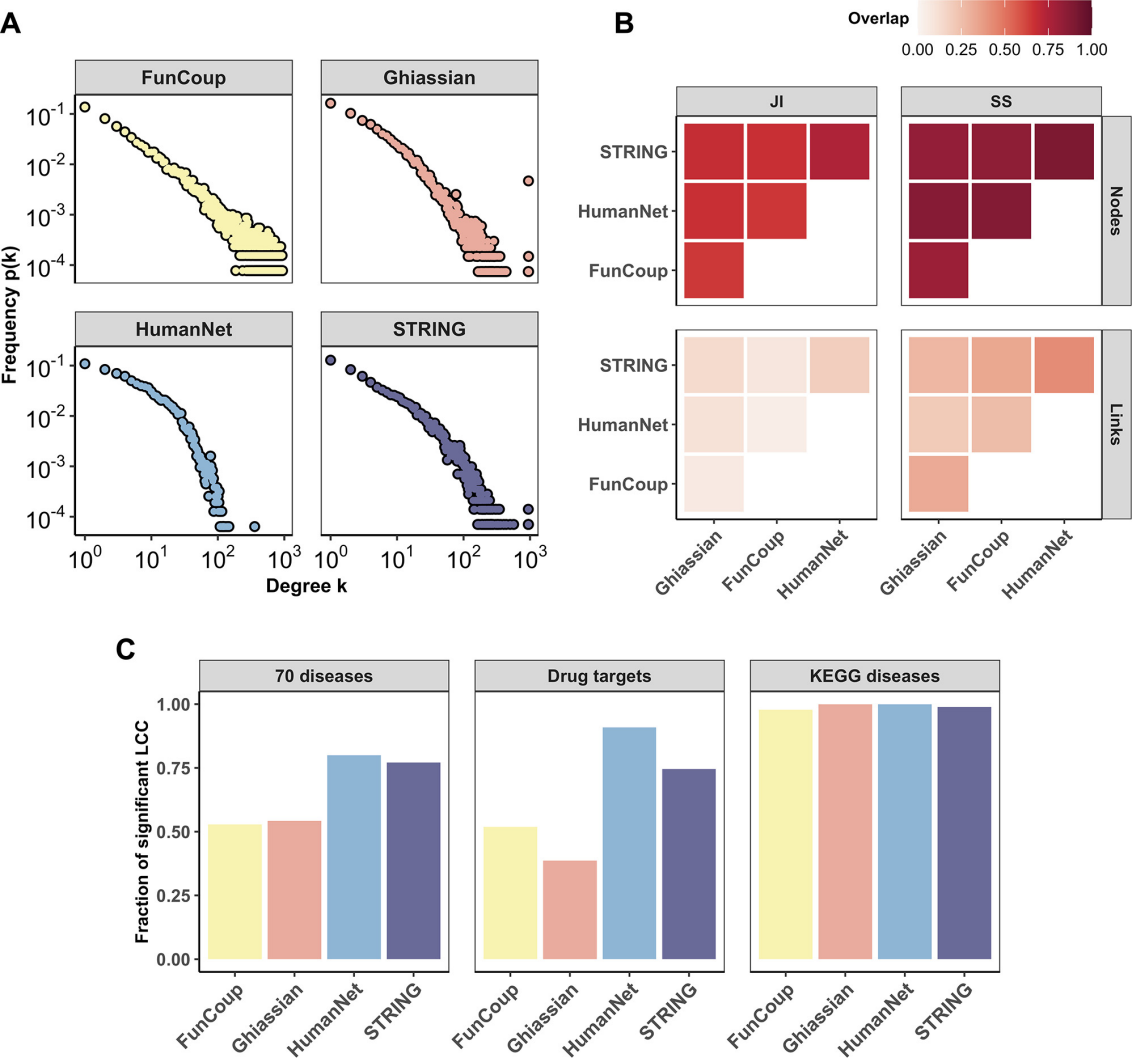
Properties of the four interactomes employed in this study. (**A**) Degree distribution $p( k )$ of the whole Ghiassian interactome and of the high confidence interactions in FunCoup, HumanNet and STRING. (**B**) Similarity between the networks expressed as Jaccard index (JI) and Szymkiewicz–Simpson (SS) coefficient for nodes and links. (**C**) Fraction of gene sets with significant LCC size for the 70 diseases, Drug targets and KEGG diseases in the different networks. LCC size was considered significant if the permutation-based FDR was below 0.05.

Four human interactomes were used in the following analysis: the Ghiassian network and the high confidence parts of FunCoup, HumanNet and STRING. All networks exhibited a scale-free topology (Figure 2A) and their sizes and topological properties are summarized in Supplementary Table S5. When testing the size of the largest connected component (LCC) of each gene set we observed that a large fraction (0.39–1) had LCCs significantly larger than expected by chance (Figure 2C). The remaining fraction of genes in each set (0.01–0.61) suggests that the networks connecting disease genes are often incomplete, or/and that the set of genes associated with a certain disease represent several unrelated mechanisms.

The differences seen between the networks may be attributed to their distinct use of sources of interactions, with HumanNet and STRING retrieving the highest proportion of disease-gene and drug-target associations, possibly due to their extensive use of curated database content and text-mining from PubMed Central articles. With the goal to produce a data-driven network, FunCoup does not directly fetch any disease-related data, which may be the reason why it has a smaller proportion of gene set LCCs that are significantly more connected than by chance. We also measured how similar the networks are in terms of common nodes and links (Figure 2B). HumanNet and STRING are the most similar networks, with STRING including about half of the links present in HumanNet. FunCoup has a considerably higher density of interactions than the other networks (3–6 times), which gives it the lowest Jaccard Index overlap for links.

For each of the four interactomes and the three collections of gene sets, each method was run, generating 12 overall results for the 4 × 382 runs per method. The distribution of produced modules per method is shown in Supplementary Figure S1. Because we are interested in connecting as many seeds as possible, only the largest predicted module by each method was taken into account for further evaluation. The largest predicted module was on average at least one order of magnitude larger than the second largest one (Supplementary Figure S2).

The benchmark only considers modules composed of at least one seed and one connector gene, and the fraction of gene sets for which each method found such a module ranged between 0.36 and 1.0 (Supplementary Table S6). With its default configuration, DIAMOnD includes 200 connectors and thus always produces such modules. On the other hand, methods such as TOPAS and SCA sometimes found relatively few such modules, with the lowest fraction of 73% for Drug targets using HumanNet. However, as shown in Figure 2C, these gene sets are already well linked in HumanNet, hence no extra connectors are needed. TOPAS, SCA and ROBUST exhibit a similar behavior for KEGG diseases in STRING. From the evidence channel ‘curated knowledge’, STRING assigns high confidence to links between genes in the same KEGG pathway, which lets TOPAS, SCA and ROBUST retrieve all seeds without any connectors. More details on the network properties of the predicted modules are reported in Supplementary Figure S3.

### Assessment of Seed Recovery Rate and Seed to Connector Ratio

**Figure 3.**
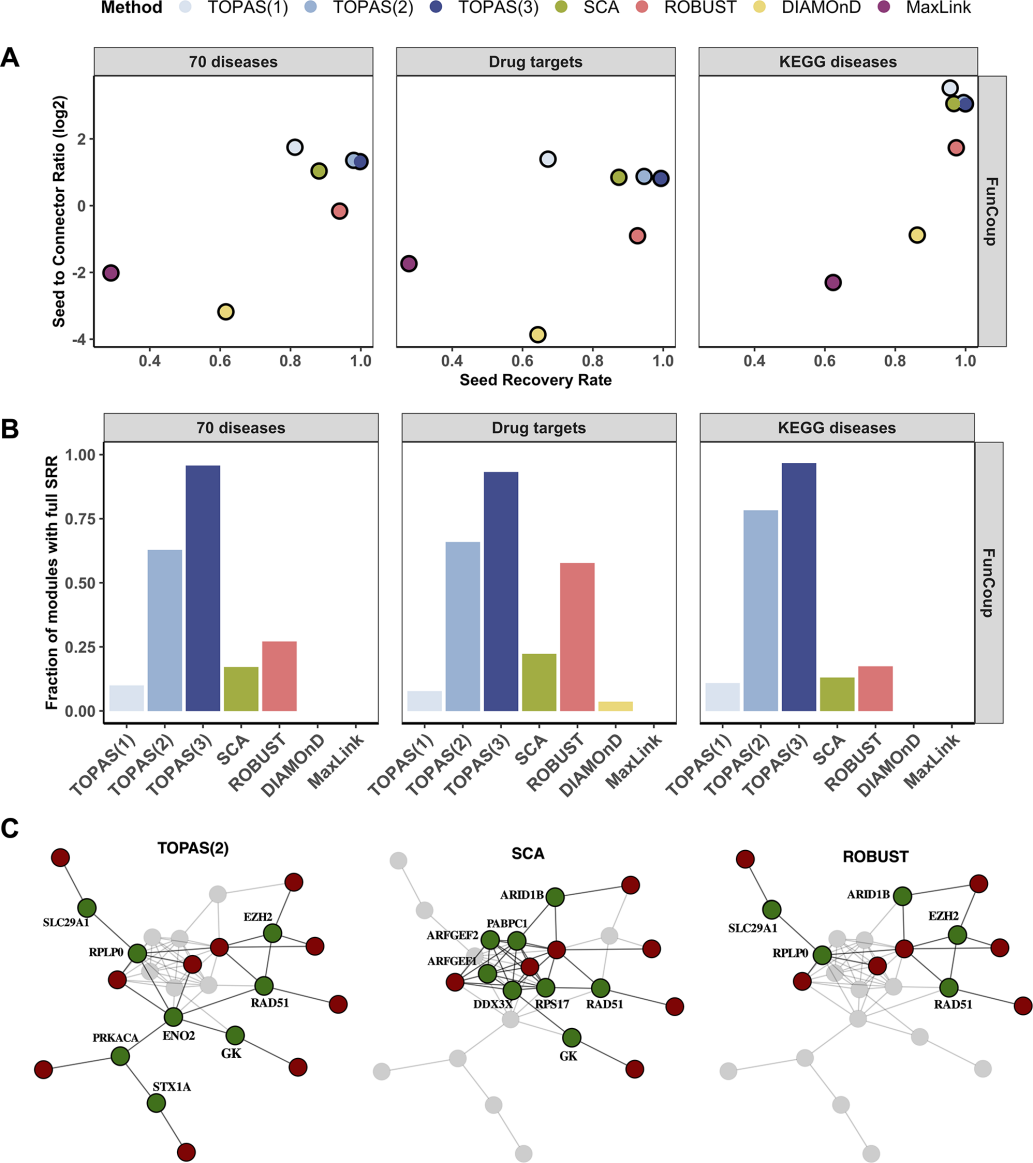
Seed Recovery Rate and Seed to Connector Ratio summary in FunCoup. (**A**) Average Seed to Connector Ratio versus Seed Recovery Rate. (**B**) Ratio of modules with full Seed Recovery Rate. The best performance is closest to the top right corner. (**C**) The drug target module for ‘DB00276’ as it is inferred by TOPAS(2), SCA and ROBUST with FunCoup. The whole network represents the union of vertices and edges inferred by the three methods. Nodes that do not belong to each method's specific prediction are shown in faint gray. Seed and connector genes are shown in red and green, respectively.

The performance of the studied methods can not be assessed in terms of sensitivity and specificity since the ground truth of disease genes, of drug targets and disease pathways genes, is unknown. Instead, we compared TOPAS to all other methods in terms of SRR and SCR. SCR, the average number of seeds per connector, reveals if an excessive amount of connectors was used. The performances of the investigated methods are summarized in Figure 3A as a combination of average SRR and SCR with FunCoup as the underlying network. TOPAS tends to have the best combined performance, followed closely by SCA. The same relationship is observed when using the other three networks (Supplementary Figure S4). In most cases, TOPAS has the highest SRR, with TOPAS(3) hitting full recovery rate for 97% of KEGG diseases in FunCoup (Figure 3B). At the lower end, MaxLink and DIAMOnD tend to almost never reach a full SRR. A few exceptions exist where MaxLink reaches maximally 12% for Drug targets in HumanNet, and DIAMOnD 33% for KEGG diseases in STRING (Supplementary Figure S5). After TOPAS, SCA and ROBUST reach a minimum of 0.69 SRR for 70 diseases in HumanNet and 0.93 for Drug targets in FunCoup. These methods do not reach near 100% SRR as TOPAS, with the exception of ROBUST using HumanNet. The complete distributions of SRR and SCR are shown in Supplementary Figures S6 and S7.

The SCR ensures that reaching a high seed coverage by massive inclusion of connectors is penalized, and avoids trivial solutions like using the entire network which will give a high SRR. Overall, TOPAS(3) and SCA show the highest SCR in all scenarios, never below 1.76 and 1.78 seeds per connector. In contrast, ROBUST, MaxLink and DIAMOnD predict more connectors than seeds in most of the cases with a minimum SCR of 0.5, 0.09 and 0.07, respectively.

As an example of disease modules found by top performing methods, Figure 3C shows the drug target module for DB00276 as inferred by TOPAS(2), SCA, and ROBUST using FunCoup. TOPAS(2) retrieved all 10 seeds (AR, ATXN2, MTOR, AOX1, GMNN, RAD52, SLC22A1, TOP2A, DRD1, KCNH2) and eight connectors representing new potential drug targets, three of which were uniquely predicted (STX1A, PRKACA, ENO2). SCA retrieved seven seeds and eight connectors, five of which were unique (RPS17, PABPC1, DDX3X, ARFGEF2, ARFGEF1), while ROBUST retrieved seven seeds and five connectors, none of which were unique. We note that DIAMOnD connected six seeds in a final module of 206 genes whereas MaxLink yielded a module of two seeds only (not shown).

### Consistency between methods

**Figure 4.**
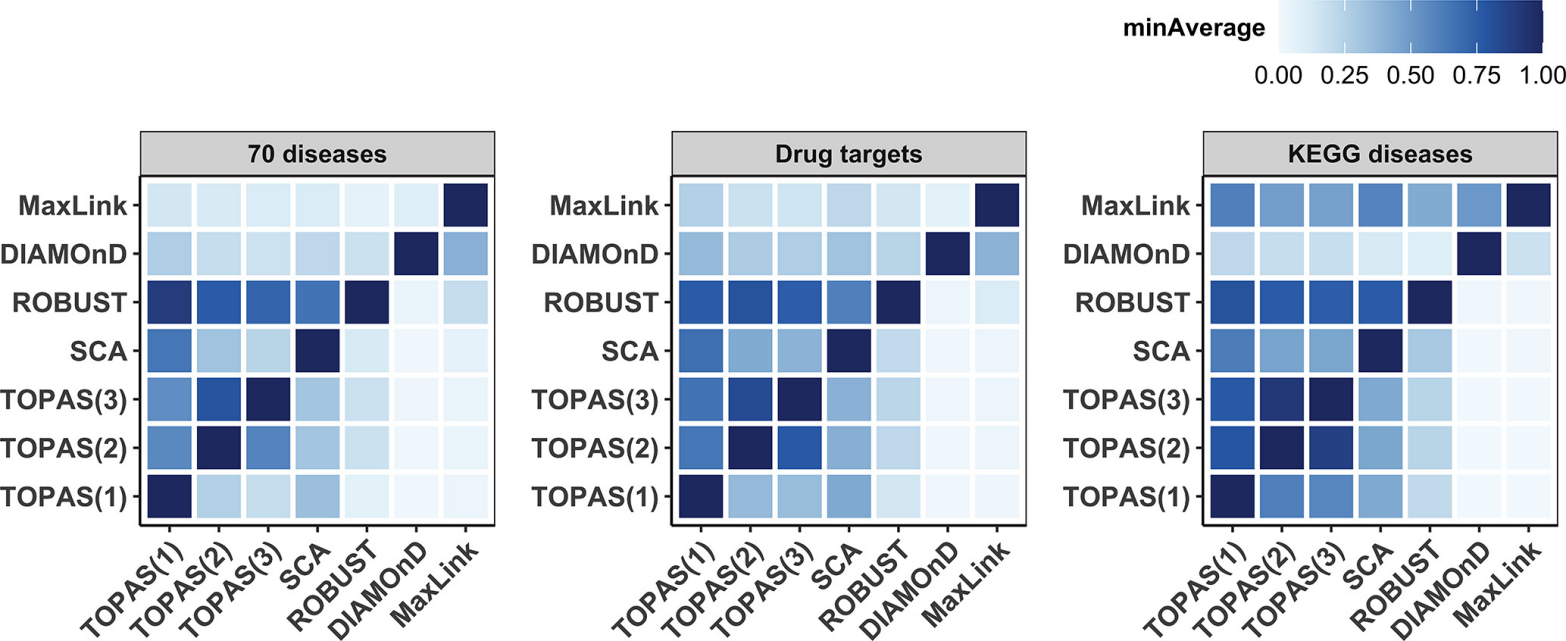
Consistency between methods. The heatmap shows the minimum average (minAverage) overlap value of connector genes between predicted modules from all combinations of methods throughout the four interactomes. Because the overlap is normalized by the number of connectors in the method on the x-axis, the matrix is asymmetric.

By examining the overlap between the modules identified by the different methods, we evaluated the benchmarked approaches in terms of their inference similarity. In Figure 4 we focus on the selection of the connector genes and have summarized the analysis by computing the average connector overlaps in all combinations of methods. We then picked the minimum average overlap of the four interactomes in order to show the maximum dissimilarity. The resulting matrix is asymmetric because the overlap is divided by the number of connectors predicted by either method. Overall, TOPAS(2) and TOPAS(3) have the largest overlap at 0.92 for KEGG diseases, which is due to the fact that most of the seeds do not lie further than two nodes apart in the networks. This means that most of the time allowing a maximum of 2 connectors between seeds is enough to reach the top seed coverage. The second largest overlap was found from ROBUST to TOPAS(1) at 0.90 for 70 diseases. However, going from TOPAS(1) to ROBUST, the highest overlap here was only 0.07, indicating that TOPAS(1) uses much fewer connectors in its modules. SCA, which also yields few connectors, has a similar asymmetric overlap with ROBUST, of 0.76 when going from ROBUST to SCA but only 0.29 the other way. DIAMOnD has very low overlap with TOPAS, SCA and ROBUST (0–0.36) but higher with MaxLink at 0.51 for KEGG diseases. The averaged JIs are reported in Supplementary Table S7 and the JI and SS distribution is shown for separate networks in Supplementary Figure S8.

### Consistency between networks

**Figure 5.**
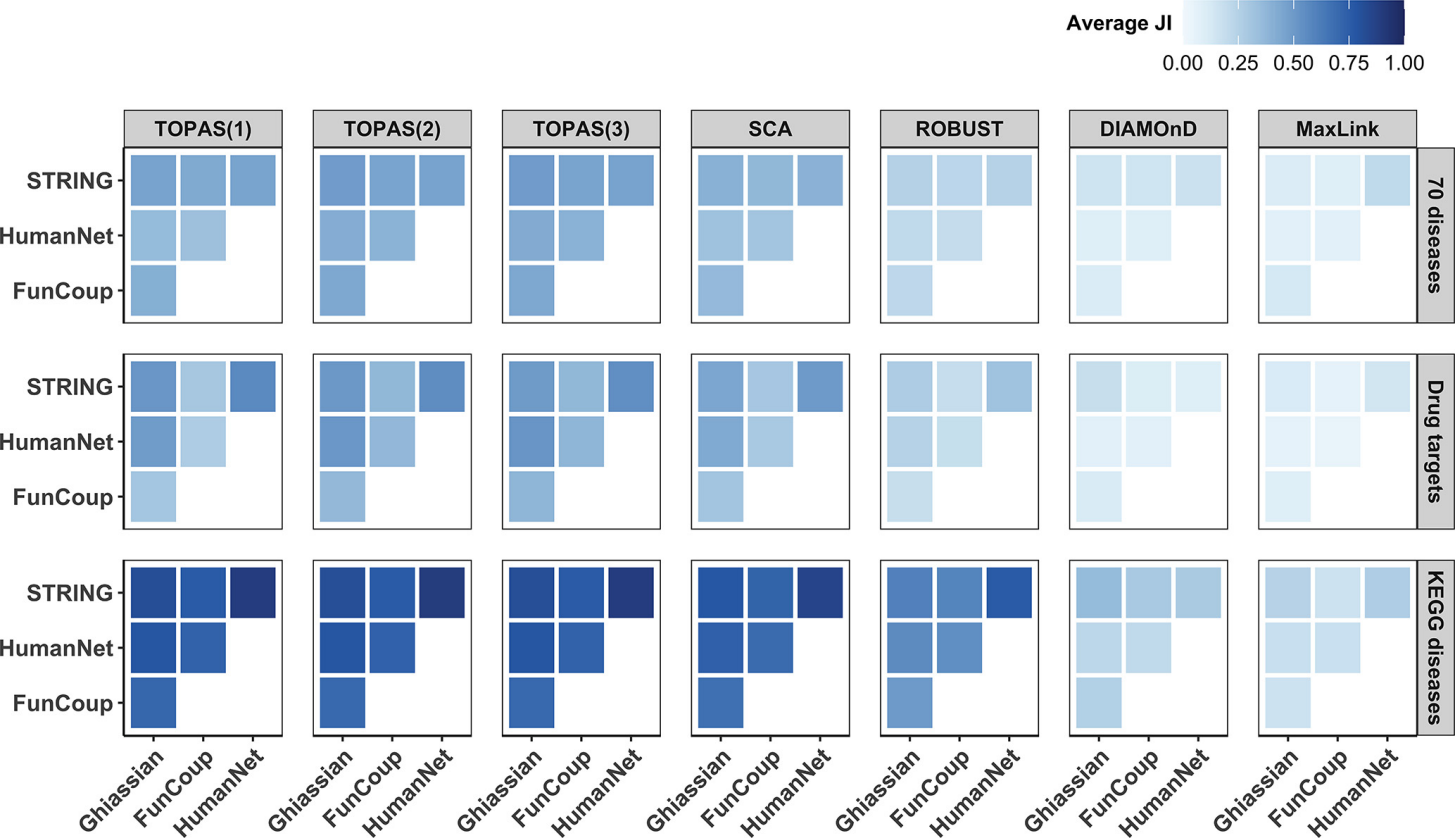
Consistency between networks. The heatmap shows the average Jaccard Index (JI) between modules predicted with the same gene set using different underlying networks.

All methods presented so far rely on a network of interactions. Despite the fact that none of the four interactomes used in this study share more than half of their links with each other (Figure 2B), we believe that a seed module detection algorithm should be designed to output the most similar answers regardless of the underlying network in use. In Figure 5 we report the method consistency between networks as the average module JI for all combinations of networks. All methods show higher module consistency between networks when KEGG diseases are used as seeds. This is potentially due to the higher connectivity of these gene sets as shown earlier. Notably, TOPAS, SCA and ROBUST have the largest consistency among all methods. Despite the differences in the underlying algorithms, these methods have the same objective: to retrieve the maximum number of seed genes and therefore should behave more similarly. Overall, TOPAS shows the highest averaged consistency between networks (Supplementary Table S8). In contrast, DIAMOnD and MaxLink stand out by inferring more dissimilar modules throughout the networks in use. The full JI distribution is shown in Supplementary Figure S9.

### Biological relevance of predicted disease modules

To assess the biological relevance of seeds and connectors in the predicted disease modules, we used their annotated pathway terms. These terms would be strongly biased in the KEGG diseases category, hence we only analyzed the 70 diseases and Drug targets. Given that experiment-derived gene sets often reflect many impacted pathways (37), we wanted to maximize the coverage of functions in the predicted module as efficiently as possible. Supplementary Figure S10 shows that TOPAS captured the largest pathway term diversity, followed closely by SCA. ROBUST yielded markedly lower diversity, at least for the 70 diseases, and DIAMOnD and MaxLink even less.

**Figure 6.**
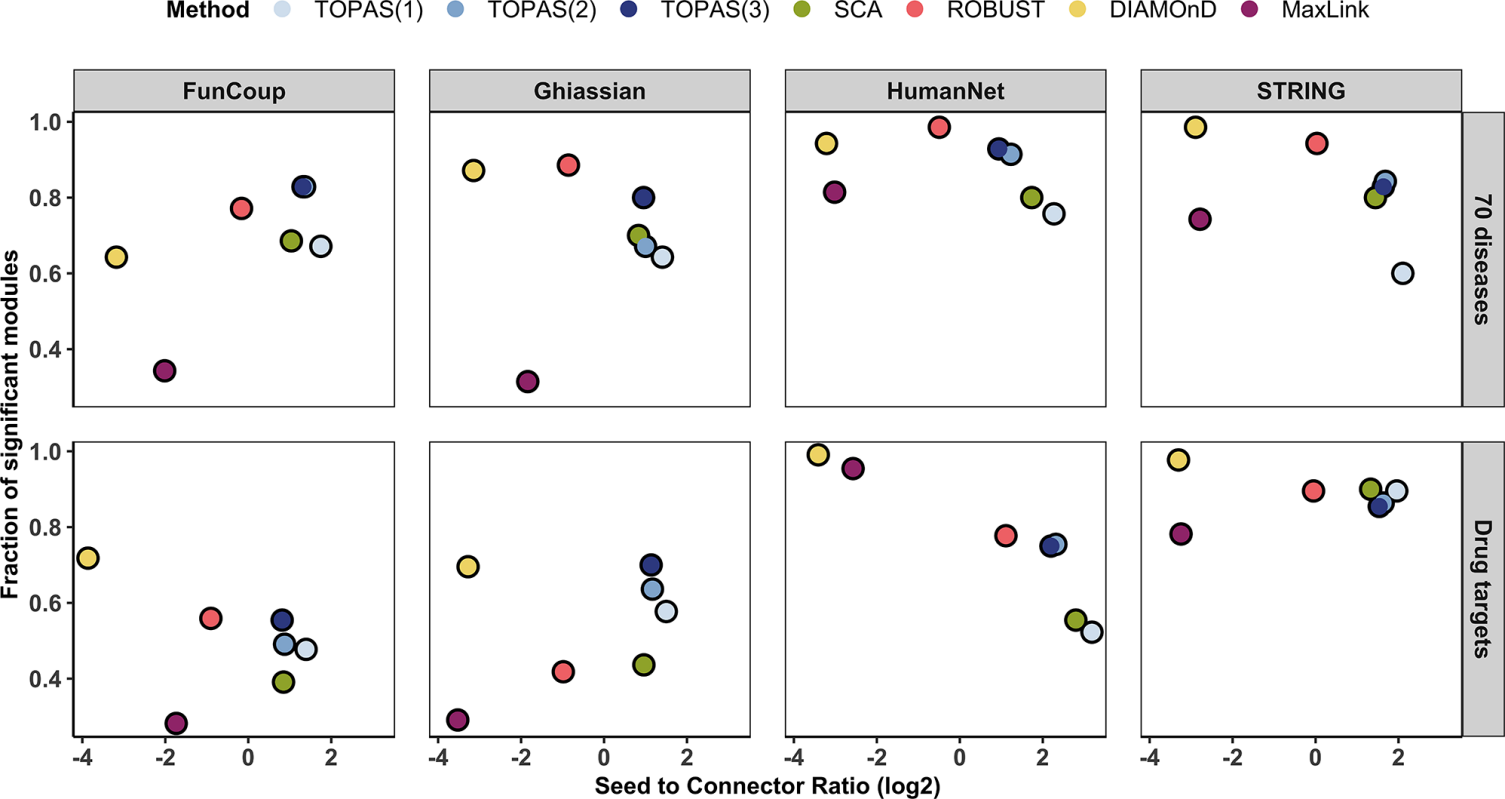
Biological relevance of seeds and connectors. Average Seed to Connector Ratio versus fraction of gene sets that have a significant (FDR < 0.05) Jaccard index overlap between the pathway terms in the seed and connector genes after subclustering the predicted disease modules.

To evaluate the biological relevance of the identified connector genes only, we analyzed their pathway term overlap with the seed genes. In order to increase the sensitivity of the analysis, we used Infomap (36) to separate modules into more closely related subsets of genes. With respect to the fraction of gene sets that have a significant overlap in at least one cluster (submodule) we found no clear overall winner (Figure 6). We then employed a combination of the fraction of significant overlaps and the average SCR to normalize for the size of the prediction. In this scenario, TOPAS was among the top methods in most cases, predicting biologically relevant modules yet selecting the fewest number of connectors.

**Figure 7.**
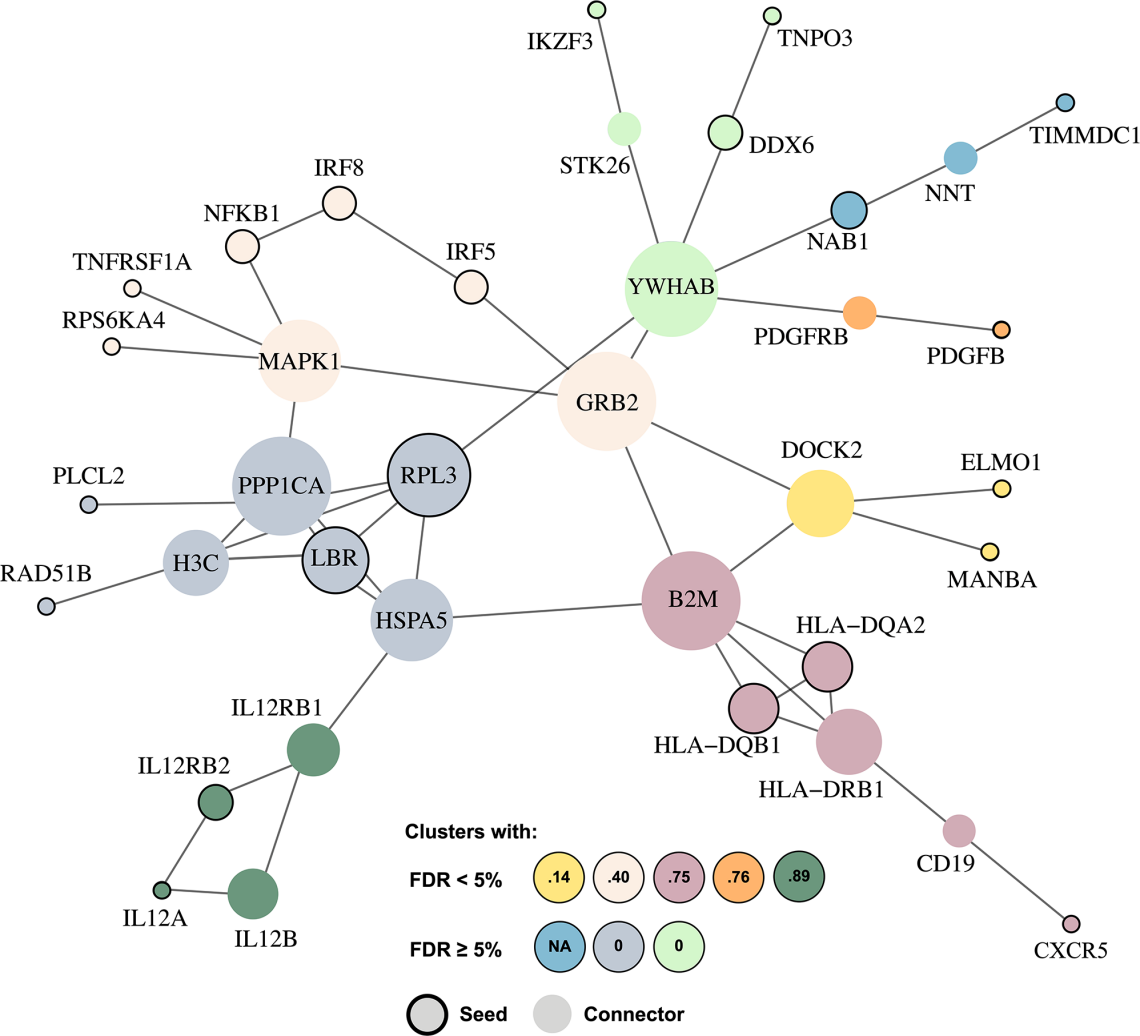
The clusters in the disease module for *Biliary liver cirrhosis* inferred by TOPAS(2) using FunCoup. The different node colors correspond to subclusters of the resulting disease module. Seed genes have a black border, while the connectors have none. The size of the nodes is proportional to the degree of connectivity. Biological relevance in terms of Jaccard index (JI) for KEGG pathway term overlap between seeds and connectors of the same subcluster is depicted at the bottom of the image as circles with colors corresponding to subclusters and numbers depicting the corresponding JI. Five out of eight subclusters show a statistically significant overlap at FDR < 5%.

In Figure 7 we report the disease module for *Biliary liver cirrhosis* as an example of a prediction by TOPAS(2) using FunCoup. The module is composed of 37 nodes, including 22 seeds and 15 connectors, and 49 edges. Infomap retrieved eight clusters, five of which had a significant (FDR < 5%) overlap of pathway terms in the aforementioned analysis. For one of the three resulting clusters it was impossible to run a significance test, due to absence of NAB1 and TIMMDC1 in any of the KEGG pathways. STK26, IKZF3 and PLCL2 were also absent from KEGG and this may have influenced the analysis for the other two clusters.

The highest JI (0.89) was achieved for the cluster containing four IL12-related genes (gene names IL12*). The IL-12 family of cytokines is crucial for immune response and has been shown to be involved in various autoimmune disorders (38). The two seed genes in this cluster are the cytokine subunit IL12A and its receptor subunit IL12RBA, previously implicated in the primary biliary cirrhosis (39), while the two detected connectors consist of other subunits for the IL12 cytokine and its receptor. This module supports the proposed autoimmune etiology of biliary cirrhosis (40). The cluster with the second highest JI (0.76) contributes the PDGF-receptor to the PDGF protein seed. There is evidence from literature supporting the implication of the PDGF-receptor in liver cirrhosis (41). At JI of 0.75 the cluster containing HLA seed genes is extended by an additional HLA connector, the DRB1. Additionally, B2M, a component of the HLA class I molecule and a potential biomarker for liver cirrhosis (42), has been identified as a connector, as well as CD19, a marker for B-lymphocytes. The involvement of B-lymphocytes in autoimmune diseases in general and biliary cirrhosis in particular has been extensively described in the literature (43). The two remaining significant subclusters at lower JI (0.4 and 0.14) contribute with other biologically relevant connectors such as MAPK (44), a putative regulator of the immune response in liver cirrhosis and DOCK2 involved in immune inflammatory processes such as lymphocyte activation and migration (45). Altogether, this demonstrates the biological relevance of the results produced by TOPAS as well as its utility for extending the knowledge of molecular disease implications and etiology.

### Runtime

**Figure 8.**
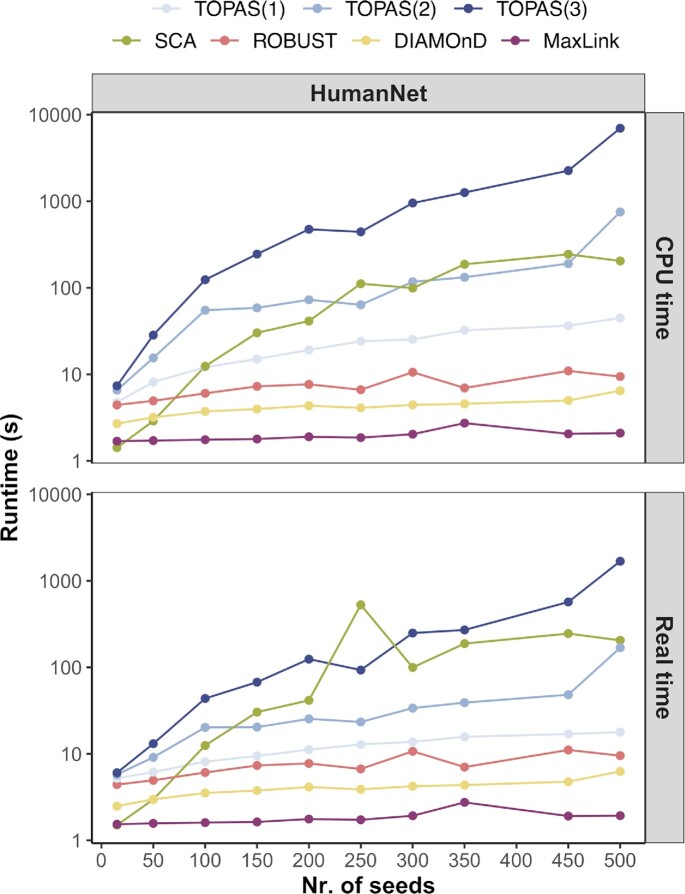
Runtime of disease module finding methods. All methods were run on a machine with four cores and eight threads. The runtime was displayed as seconds versus number of input seeds.

We performed scalability tests for each of the benchmarked disease module finding methods. To detect trends on the runtime over all data in use, we show the runtime versus the size of the input (Figure 8). The analysis was done on HumanNet with KEGG diseases as input. We observed a sublinear relationship with the number of input seeds for DIAMOnD, MaxLink, and ROBUST. TOPAS and SCA scaled approximately quadratically with the size of the input. However, TOPAS and SCA produced a result in a few seconds or minutes for the majority of gene sets. TOPAS allows for parallelization during the computation of all shortest paths between seeds, which can vastly reduce the elapsed time when it analyzes large gene sets.

## DISCUSSION AND CONCLUSION

We have presented a new network-based iterative approach TOPAS to detect disease modules in a top-down fashion, and showed that it outperforms existing methods in terms of Seed Recovery Rate (SRR), Seed to Connector Ratio (SCR) and consistency during disease module detection.

We tested the interconnectivity of 382 disease gene sets and show that a big percentage of genes annotated with the same disease are part of a largest connected component whose size is statistically significant. This comes in support of the mounting evidence that proteins, when associated with the same disease, collaborate in the dysfunction of the system to which they belong. On the other hand the remaining percentage of unconnected genes indicates that disease modules are still incomplete. This may be due to the incompleteness of the network, but also to the heterogeneity of the disease gene set. For instance, the genes may represent a number of independent mechanisms, or include unrelated driver and passenger disease genes (i.e. genes causing the disease and those affected by it). Therefore, methods like TOPAS are needed to fill this knowledge gap by identifying optimal disease modules to promote further understanding of the molecular mechanisms of the disease.

We benchmarked TOPAS against a selection of existing network-based disease module detection methods that rely exclusively on prior disease gene sets. Specifically, we compared TOPAS to bottom-up methods such as SCA, DIAMOnD, and MaxLink, as well as to ROBUST, a top-down strategy. Despite the fact that the method DOMINO performed well in an earlier evaluation (16,17), we have not included DOMINO in the study because it has a different goal than the other included methods: it partitions the seed genes into optimal submodules, rather than trying to retrieve one optimal disease module. Because of this, DOMINO produces much more separated modules than the other methods, with no clear largest module to include in the analysis (Supplementary Figures S1 and S2).

The standard metrics to evaluate the performance of a classifier such as sensitivity and specificity could not be employed in this study since the ground truth of disease genes is unknown. Instead, we compared TOPAS to all other approaches in terms of SRR and SCR. We demonstrated that TOPAS consistently has the highest SRR and achieves, unlike any other method in this study, near-100 percent recovery rate of seeds in a single module. In contrast, DIAMOnD and MaxLink are unsuitable for this task since their aim is to unlimitedly expand the immediate local network neighborhood of the input seeds, and indeed showed poor performances in our benchmark. An alternative to measure the performance of the methods would be to subsample multiple times the input seed gene sets and count the number of left out seeds in the final module. However, because disease gene sets are heterogeneous and seed genes may lie in unconnected areas of the network, such a cross validation based analysis would not be appropriate to assess the performances of TOPAS, SCA, and ROBUST whose objective is to exclusively find the most suited nodes to connect the input seed genes.

We believe that a seed module discovery technique should be consistently producing the most similar results regardless of the underlying network. We showed that TOPAS, followed by SCA and ROBUST, reaches the highest consistency of all evaluated methods between the used networks. DIAMOnD and MaxLink stand out because they infer the most diverse modules, which is not surprising given their poor SRR and SCR. Those two methods, while good at finding new related genes to a seed set, do not appear to be well suited for building concise and consistent disease modules.

We utilized annotated pathway terms to assess the biological relevance of seeds and connectors in predicted disease modules. Given that experiment-derived gene sets frequently represent many perturbed pathways (37), we aimed at optimizing function coverage in the predicted module as effectively as possible and showed that TOPAS captures the most pathway term variety despite using few connector genes. To assess the biological relevance of the predicted connectors only, we first clustered the modules into tighter submodules and then examined their pathway term overlap with the seed genes. Here TOPAS demonstrated competitive performance.

The majority of previous methods extend disease modules by iteratively adding connector genes from the bottom up, while the top-down approach had only been explored by the application of Steiner trees as in ROBUST. TOPAS is a novel top-down strategy that outperforms ROBUST and overcomes the shortcomings that are inherent in bottom-up methods, resulting in poor SRR and SCR as exhibited by DIAMOnD and MaxLink. TOPAS builds disease modules by starting from a full seed network, thus having an overall picture of the interconnectivity of the seed genes in the input. The advantage is that the disease module is not expanded based on decisions taken on local areas of seed subnetworks, but rather pruned down from the largest network of seeds possible. In such a way, we can (i) run Random Walk with Restart to find optimal paths in the whole network and guarantee the functional relationship of selected connectors with the input seeds, and (ii) let the user decide the maximum number of connectors between seeds to overcome the limitation of direct neighbors imposed in SCA.

TOPAS was tested on disease-related gene sets to find biologically relevant disease modules, but may be employed with any collection of functionally related molecular units as long as a comprehensive network of their interactions exists and there is supporting evidence in favor of maximizing the SRR.

## DATA AVAILABILITY

The R package of TOPAS is available at https://bitbucket.org/sonnhammergroup/topas.

## Supplementary Material

lqac093_Supplemental_File
