# Supplementary material for: TOPAS, a network-based approach to detect disease modules in a top-down fashion
Source: NAR Genom Bioinform. 2022 Nov 29;4(4):lqac093. doi: 10.1093/nargab/lqac093 (PMC9706483; doi:10.1093/nargab/lqac093)
Supplement: lqac093_Supplemental_File [file lqac093_supplemental_file.pdf]

Supplementary Materials

TOPAS, a network-based approach to detect disease modules in a top-down fashion.

Supplementary Tables

Supplementary Table 1. Seed genes overview. Each gene set of the 70 diseases, Drug targets or KEGG diseases in this study has an identifier. We show the original number of the seed genes and the size of the mapped seed module per network. The size of the seed module varies accordingly with network coverage and the gene vocabulary in use (*i.e.* a *Gene ID* or a *Gene Name* can map to multiple *Ensembl* gene and protein identifiers and the other way around). The largest amount of missing genes (some of which is untranslated) in the KEGG diseases benchmark refer to non coding DNA, most of which are part of the pathway *"MicroRNAs in cancer (hsa05206)"*.

		FunCoup	Ghiassian	HumanNet	STRING
**70 diseases (1536)**	**Network seeds**	1302	1536	1464	1474
	**Missing**	234	0	72	62
	**Untranslated**	7	0	0	4
**Drug targets (764)**	**Network seeds**	626	732	732	756
	**Missing**	138	32	32	8
	**Untranslated**	15	17	17	1
**KEGG diseases (3531)**	**Network seeds**	2724	2947	3036	3006
	**Missing**	807	584	495	525
	**Untranslated**	172	0	0	173

Supplementary Table 2. 70 diseases seeds overview. Each row of the Supplementary Table shows the ID of the disease under study followed by the original and the mapped number of seed genes in every network. For more details, refer to description in Supplementary Table 1.

Target	seeds	FunCoup	STRING	HumanNet	Ghiassian	Target	seeds	FunCoup	STRING	HumanNet	Ghiassian
adrenal gland diseases	18	10	18	18	18	glomerulonephritis	18	14	17	18	18
alzheimer disease	29	24	27	24	29	gout	13	10	13	13	13
amino acid metabolism inborn errors	52	50	53	52	52	graves disease	13	10	12	11	13
amyotrophic lateral sclerosis	21	20	20	21	21	head and neck neoplasms	35	29	33	31	35
anemia aplastic	21	20	21	21	21	hypothalamic diseases	23	22	23	23	23
anemia hemolytic	29	27	29	29	29	leukemia b-cell	17	13	14	17	17
aneurysm	15	14	15	15	15	leukemia myeloid	43	41	41	42	43
arrhythmias cardiac	30	21	28	28	30	lipid metabolism disorders	50	48	52	49	50
arterial occlusive diseases	44	40	43	40	44	liver cirrhosis	24	23	23	24	24
arteriosclerosis	38	34	37	34	38	liver cirrhosis biliary	23	22	22	23	23
arthritis rheumatoid	42	34	37	39	42	lung diseases obstructive	40	34	36	38	40
asthma	37	32	34	35	37	lupus erythematosus	75	58	63	62	75
basal ganglia diseases	45	39	45	44	45	lymphoma	24	20	25	23	24
behcet syndrome	13	9	11	9	13	lysosomal storage diseases	45	43	45	45	45
bile duct diseases	31	29	30	31	31	macular degeneration	44	32	42	41	44
blood coagulation disorders	40	36	40	40	40	metabolic syndrome x	14	11	14	14	14
blood platelet disorders	26	22	26	26	26	motor neuron disease	31	30	30	31	31
breast neoplasms	40	33	39	38	40	multiple sclerosis	69	59	65	66	69
carbohydrate metabolism inborn errors	77	72	78	73	77	muscular dystrophies	36	34	36	36	36
carcinoma renal cell	18	17	18	17	18	mycobacterium infections	22	21	22	22	22
cardiomyopathies	50	47	50	50	50	myeloproliferative disorders	19	18	19	18	19
cardiomyopathy hypertrophic	22	20	22	22	22	nutritional and metabolic diseases	599	514	591	569	599
celiac disease	36	27	36	32	36	peroxisomal disorders	20	20	20	20	20
cerebellar ataxia	30	26	29	30	30	psoriasis	54	47	48	48	54
cerebrovascular disorders	47	35	45	42	47	purine-pyrimidine metabolism inborn errors	16	13	16	16	16
charcot-marie-tooth disease	27	25	27	27	27	renal tubular transport inborn errors	34	23	34	33	34

**colitis ulcerative**	56	46	52	52	56	**sarcoma**	25	22	25	25	25
**colorectal neoplasms**	42	38	40	40	42	**spastic paraplegia hereditary**	20	18	19	20	20
**coronary artery disease**	31	27	30	28	31	**spinocerebellar ataxias**	28	24	27	28	28
**crohn disease**	72	53	67	70	72	**spinocerebellar degenerations**	30	26	29	30	30
**death sudden**	19	18	18	19	19	**spondylarthropathies**	18	15	17	16	18
**diabetes mellitus type 2**	73	63	69	70	73	**tauopathies**	35	29	33	30	35
**dwarfism**	26	22	25	26	26	**uveal diseases**	17	13	15	13	17
**esophageal diseases**	24	20	21	21	24	**varicose veins**	20	16	20	19	20
**exophthalmos**	13	10	12	11	13	**vasculitis**	15	11	13	11	15

Supplementary Table 3. Drug targets overview. Each row of the Supplementary Table shows the ID of the drug under study followed by the original and the mapped number of seed genes in every network. For more details, refer to description in Supplementary Table 1.

Target	seeds	FunCoup	STRING	HumanNet	Ghiassian	Target	seeds	FunCoup	STRING	HumanNet	Ghiassian
**DB00131**	12	10	12	11	11	**DB00882**	28	19	30	27	27
**DB00142**	19	15	18	19	18	**DB00887**	11	7	11	11	7
**DB00166**	11	11	11	11	11	**DB00909**	12	7	12	12	7
**DB00170**	13	12	13	13	13	**DB00917**	10	4	9	10	10
**DB00179**	19	14	20	19	18	**DB00920**	16	9	18	16	16
**DB00188**	14	13	15	14	14	**DB00928**	14	12	15	14	13
**DB00194**	10	6	10	10	9	**DB00931**	10	10	10	10	10
**DB00197**	10	7	10	10	8	**DB00933**	18	15	19	18	18
**DB00203**	14	6	14	14	13	**DB00934**	24	15	25	24	24
**DB00206**	11	11	11	11	11	**DB00935**	17	9	15	16	17
**DB00215**	11	8	12	10	11	**DB00967**	16	11	18	16	16
**DB00239**	18	11	18	18	17	**DB00968**	15	15	15	15	15
**DB00245**	20	12	22	19	20	**DB00975**	14	7	14	13	12
**DB00246**	34	23	34	32	32	**DB00977**	11	9	12	11	11
**DB00247**	24	17	22	23	24	**DB00985**	16	10	17	15	16
**DB00248**	17	10	16	17	17	**DB00988**	19	18	19	19	19
**DB00251**	19	12	19	17	18	**DB00999**	10	6	10	10	6
**DB00255**	31	23	31	31	30	**DB01021**	10	6	10	10	6
**DB00257**	48	35	51	47	47	**DB01026**	16	13	17	16	16
**DB00259**	11	5	11	11	7	**DB01062**	11	9	11	10	11
**DB00268**	11	7	10	11	11	**DB01064**	13	10	13	11	12
**DB00273**	11	6	11	11	6	**DB01069**	24	15	26	23	24
**DB00276**	10	10	11	10	10	**DB01074**	10	8	10	9	9
**DB00283**	23	14	25	22	23	**DB01092**	12	12	13	12	12

**DB00285**	10	7	10	10	10	**DB01100**	27	20	28	27	27
**DB00289**	18	15	18	18	18	**DB01103**	24	17	25	24	24
**DB00310**	10	6	10	10	6	**DB01104**	20	13	21	19	20
**DB00317**	83	71	81	74	82	**DB01110**	37	24	38	37	36
**DB00320**	18	12	17	18	18	**DB01113**	11	8	12	11	11
**DB00321**	31	21	32	30	31	**DB01114**	18	11	20	17	18
**DB00328**	11	8	12	10	10	**DB01115**	19	16	19	19	18
**DB00334**	30	21	31	29	29	**DB01118**	36	26	38	35	35
**DB00342**	23	18	24	23	23	**DB01127**	39	26	40	38	38
**DB00353**	15	9	13	15	15	**DB01129**	10	8	11	10	9
**DB00363**	37	23	36	35	34	**DB01136**	20	12	20	19	20
**DB00368**	26	22	28	25	26	**DB01142**	25	15	27	24	25
**DB00370**	14	10	16	14	14	**DB01144**	11	6	11	11	6
**DB00390**	15	14	16	15	15	**DB01149**	13	11	16	13	13
**DB00391**	13	8	13	13	10	**DB01151**	22	17	25	22	22
**DB00396**	11	8	11	10	11	**DB01160**	10	4	9	10	10
**DB00398**	126	111	122	115	124	**DB01173**	17	10	19	17	17
**DB00406**	34	24	35	34	33	**DB01176**	13	8	14	13	13
**DB00408**	29	19	29	29	28	**DB01177**	16	15	18	16	16
**DB00413**	11	7	11	11	11	**DB01182**	14	11	14	12	14
**DB00420**	25	17	27	24	25	**DB01184**	17	9	18	17	17
**DB00433**	28	21	29	27	28	**DB01186**	19	12	18	19	19
**DB00434**	25	16	26	25	25	**DB01194**	11	6	11	11	6
**DB00443**	11	10	12	11	11	**DB01200**	23	16	22	22	23
**DB00445**	36	35	39	36	36	**DB01204**	44	40	47	42	42
**DB00450**	15	8	16	15	15	**DB01224**	36	24	33	33	34
**DB00457**	18	12	16	18	17	**DB01235**	17	16	18	17	17
**DB00458**	23	17	26	23	22	**DB01238**	36	26	35	34	34
**DB00471**	19	12	18	18	18	**DB01239**	13	11	13	13	13
**DB00472**	26	20	27	25	26	**DB01242**	24	16	26	23	24
**DB00476**	20	14	18	20	20	**DB01253**	10	5	9	10	10
**DB00477**	49	36	47	50	50	**DB01254**	126	114	124	120	124
**DB00481**	31	23	34	30	31	**DB01259**	16	16	16	14	16
**DB00482**	18	11	18	18	13	**DB01267**	29	19	29	28	28
**DB00490**	10	7	10	9	10	**DB01268**	253	224	242	235	248
**DB00502**	36	23	36	33	34	**DB01392**	20	11	17	20	19
**DB00530**	95	84	91	86	93	**DB01394**	13	11	14	12	12
**DB00540**	25	16	27	25	25	**DB01406**	16	10	17	16	15
**DB00543**	28	18	29	28	28	**DB01422**	10	10	11	10	10
**DB00549**	11	8	11	11	10	**DB01618**	13	9	15	13	13
**DB00563**	12	11	11	12	12	**DB01623**	10	7	11	10	10
**DB00566**	11	10	11	11	11	**DB02266**	10	8	10	10	10
**DB00568**	20	13	21	19	20	**DB02530**	10	8	10	10	8

**DB00571**	17	14	16	15	17	**DB02546**	17	17	17	17	17
**DB00574**	21	16	21	19	21	**DB02731**	11	7	11	11	7
**DB00575**	10	6	10	9	10	**DB03128**	10	9	10	11	9
**DB00580**	14	7	14	13	8	**DB04813**	30	20	30	30	28
**DB00586**	11	9	12	10	10	**DB04815**	13	12	14	13	13
**DB00589**	23	14	24	22	23	**DB04819**	14	9	15	14	14
**DB00590**	12	7	12	11	12	**DB04841**	30	19	31	29	29
**DB00595**	14	14	16	14	14	**DB04842**	24	19	23	23	24
**DB00604**	18	11	17	17	18	**DB04849**	83	76	84	77	83
**DB00619**	56	45	55	52	52	**DB04868**	67	60	66	64	63
**DB00623**	35	23	37	34	34	**DB04946**	23	18	23	22	23
**DB00637**	38	24	40	37	38	**DB05015**	12	12	12	12	12
**DB00656**	22	15	23	21	22	**DB05294**	108	95	105	101	105
**DB00661**	15	10	16	15	14	**DB05521**	11	8	10	9	9
**DB00668**	16	10	16	14	15	**DB06144**	27	21	26	26	27
**DB00669**	11	7	7	11	10	**DB06148**	29	18	28	29	29
**DB00675**	48	35	50	47	47	**DB06216**	30	22	31	29	30
**DB00679**	41	28	41	40	40	**DB06218**	12	6	12	12	7
**DB00692**	16	9	16	14	16	**DB06288**	11	8	10	11	11
**DB00694**	26	24	29	25	24	**DB06589**	106	94	107	102	105
**DB00695**	12	7	12	12	7	**DB06616**	189	168	189	177	186
**DB00696**	27	17	24	26	26	**DB06689**	10	8	11	10	10
**DB00703**	13	7	13	13	8	**DB06691**	10	6	11	9	9
**DB00714**	31	25	31	31	31	**DB06803**	21	20	22	21	21
**DB00715**	16	13	16	15	16	**DB06820**	21	12	21	21	20
**DB00734**	35	23	33	32	33	**DB08162**	30	30	31	29	30
**DB00738**	21	14	23	20	20	**DB08183**	44	38	43	40	42
**DB00755**	13	11	13	12	11	**DB08329**	12	6	12	12	7
**DB00756**	42	33	45	42	40	**DB08700**	152	134	147	139	149
**DB00783**	12	10	12	12	12	**DB08877**	131	120	127	122	126
**DB00793**	17	10	17	17	16	**DB08901**	11	11	11	11	11
**DB00804**	12	8	13	11	12	**DB08916**	39	34	38	33	39
**DB00808**	12	7	12	12	7	**DB09000**	13	11	13	13	13
**DB00819**	12	6	12	12	7	**DB09053**	22	21	23	22	22
**DB00822**	36	28	38	35	35	**DB09063**	34	31	35	34	34
**DB00831**	23	19	26	24	25	**DB09068**	13	10	14	12	12
**DB00834**	13	10	14	13	13	**DB09073**	30	27	31	29	30
**DB00836**	14	11	16	12	14	**DB09079**	15	15	15	15	15
**DB00841**	27	19	27	25	23	**DB09115**	13	12	13	13	12
**DB00850**	20	16	22	20	20	**DB09224**	11	9	11	11	11
**DB00852**	15	8	15	14	13	**DB09225**	34	24	34	33	33
**DB00869**	12	6	12	12	7	**DB09286**	18	11	16	17	18
**DB00878**	13	10	12	13	13	**DB09298**	13	5	13	13	9

Supplementary Table 4. KEGG diseases overview. Each row of the Supplementary Table shows the ID of the pathway under study followed by the original and the mapped number of seed genes in every network. For more details, refer to description in Supplementary Table 1.

Target	seeds	FunCoup	STRING	HumanNet	Ghiassian	Target	seeds	FunCoup	STRING	HumanNet	Ghiassian
**hsa04930**	46	44	46	46	46	**hsa05169**	202	181	199	194	191
**hsa04931**	108	99	107	105	104	**hsa05170**	212	193	211	207	195
**hsa04932**	155	147	155	148	153	**hsa05171**	232	203	230	226	219
**hsa04933**	100	94	100	99	100	**hsa05200**	531	456	527	527	505
**hsa04934**	155	124	154	152	145	**hsa05202**	193	172	190	191	187
**hsa04936**	142	122	141	140	130	**hsa05203**	204	190	201	197	196
**hsa04940**	43	37	41	41	43	**hsa05204**	69	42	63	67	50
**hsa04950**	26	22	25	25	24	**hsa05205**	205	186	201	202	193
**hsa05010**	384	346	380	364	353	**hsa05206**	310	148	162	159	158
**hsa05012**	266	245	266	249	246	**hsa05207**	212	157	189	194	174
**hsa05014**	364	333	361	343	331	**hsa05208**	223	196	217	205	209
**hsa05016**	306	280	306	287	284	**hsa05210**	86	84	86	86	85
**hsa05017**	143	136	140	140	132	**hsa05211**	69	68	69	68	68
**hsa05020**	273	253	272	253	264	**hsa05212**	76	75	76	76	75
**hsa05022**	476	429	474	454	436	**hsa05213**	58	57	58	58	57
**hsa05030**	49	47	49	49	48	**hsa05214**	75	72	75	75	71
**hsa05031**	69	65	69	68	66	**hsa05215**	97	96	97	97	97
**hsa05032**	91	69	89	91	85	**hsa05216**	37	36	37	37	36
**hsa05033**	40	36	38	40	31	**hsa05217**	63	49	63	63	55
**hsa05034**	187	172	185	182	171	**hsa05218**	72	56	72	72	68
**hsa05100**	77	75	77	74	73	**hsa05219**	41	39	41	41	41
**hsa05110**	50	50	50	49	45	**hsa05220**	76	75	76	76	74
**hsa05120**	70	68	70	68	66	**hsa05221**	67	63	67	67	67
**hsa05130**	197	175	186	193	178	**hsa05222**	92	89	91	92	91
**hsa05131**	247	234	245	241	237	**hsa05223**	72	69	72	72	71
**hsa05132**	249	240	248	247	238	**hsa05224**	147	117	147	147	135
**hsa05133**	76	64	76	75	74	**hsa05225**	168	152	165	165	154
**hsa05134**	57	54	57	55	56	**hsa05226**	149	118	146	147	136
**hsa05135**	137	130	135	134	134	**hsa05230**	70	69	70	70	68
**hsa05140**	77	66	73	72	75	**hsa05231**	98	85	84	97	89
**hsa05142**	102	93	101	100	102	**hsa05235**	89	87	89	86	88
**hsa05143**	37	28	37	35	35	**hsa05310**	31	19	28	28	29
**hsa05144**	50	41	50	48	47	**hsa05320**	53	31	50	50	46
**hsa05145**	112	106	109	109	110	**hsa05321**	65	50	63	63	65
**hsa05146**	102	91	99	100	99	**hsa05322**	136	116	131	128	124
**hsa05150**	96	60	71	88	85	**hsa05323**	93	79	89	88	88
**hsa05152**	180	151	176	172	165	**hsa05330**	38	29	35	35	37

**hsa05160**	157	128	151	156	136	**hsa05332**	42	30	38	38	42
**hsa05161**	162	149	161	160	154	**hsa05340**	38	34	36	35	36
**hsa05162**	139	122	139	138	131	**hsa05410**	90	80	90	89	83
**hsa05163**	225	198	223	221	216	**hsa05412**	77	70	77	77	70
**hsa05164**	171	145	168	166	154	**hsa05414**	96	83	95	94	88
**hsa05165**	331	290	327	328	306	**hsa05415**	203	183	202	186	197
**hsa05166**	222	211	220	217	220	**hsa05416**	60	55	57	57	59
**hsa05167**	194	172	192	192	181	**hsa05417**	215	192	214	212	204
**hsa05168**	495	275	212	237	314	**hsa05418**	139	129	136	136	133

Supplementary Table 5. Basic network properties. Number (Nr.) of nodes and edges are computed for the full network and for its Largest Connected Component (LCC).

	**FunCoup**	**Ghiassian**	**HumanNet**	**STRING**
**Nr. of nodes**	12890	13460	15677	14221
**Nr. of edges**	612276	138427	112549	180391
**Nr. of nodes in LCC**	12675	13329	14560	13741
**Nr. of edges in LCC**	612151	138356	111217	180067
**mean node degree**	95	21	14	25
**median node degree**	13	7	9	11
**mean clustering coefficient**	0,34	0,21	0,59	0,45
**mean shortest path**	4,1	4,5	5,4	4,8
**diameter**	13	13	17	15

Supplementary Table 6. Number of gene sets with at least one seed and one connector. The respective fraction to the total number of gene sets is shown within parentheses.

		TOPAS(1)	TOPAS(2)	TOPAS(3)	SCA	ROBUST	DIAMOnD	MaxLink
FunCoup	70 diseases	69 (0.99)	70 (1)	70 (1)	69 (0.99)	69 (0.99)	70 (1)	34 (0.49)
	Drug targets	181 (0.82)	218 (0.99)	219 (1)	174 (0.79)	220 (1)	220 (1)	80 (0.36)
	KEGG diseases	92 (1)	92 (1)	92 (1)	92 (1)	92 (1)	92 (1)	91 (0.99)
Ghiassian	70 diseases	70 (1)	70 (1)	70 (1)	67 (0.96)	70 (1)	70 (1)	33 (0.47)
	Drug targets	218 (0.99)	219 (1)	219 (1)	217 (0.99)	219 (1)	220 (1)	129 (0.59)
	KEGG diseases	91 (0.99)	91 (0.99)	91 (0.99)	91 (0.99)	91 (0.99)	92 (1)	92 (1)
HumanNet	70 diseases	66 (0.94)	70 (1)	70 (1)	64 (0.91)	70 (1)	70 (1)	58 (0.83)
	Drug targets	162 (0.74)	192 (0.87)	195 (0.89)	161 (0.73)	194 (0.88)	220 (1)	210 (0.95)
	KEGG diseases	82 (0.89)	85 (0.92)	85 (0.92)	82 (0.89)	84 (0.91)	92 (1)	92 (1)
STRING	70 diseases	69 (0.99)	70 (1)	70 (1)	69 (0.99)	70 (1)	70 (1)	55 (0.79)
	Drug targets	219 (1)	220 (1)	220 (1)	219 (1)	219 (1)	220 (1)	182 (0.83)
	KEGG diseases	71 (0.77)	72 (0.78)	72 (0.78)	71 (0.77)	66 (0.72)	92 (1)	92 (1)

Supplementary Table 7. Consistency between methods as averaged Jaccard-Index (JI) and Szymkiewicz–Simpson coefficient (SS). The maximum overlap between different methods is highlighted in bold.

Jaccard-Index

	TOPAS(1)	TOPAS(2)	TOPAS(3)	SCA	ROBUST	DIAMOnD	MaxLink	
**TOPAS(1)**		0,34	0,31	**0,36**	0,13	0,01	0,04	**70 diseases**
**TOPAS(2)**			0,68	0,26	0,20	0,02	0,03	
**TOPAS(3)**				0,24	0,22	0,02	0,03	
**SCA**					0,17	0,02	0,04	
**ROBUST**						0,05	0,07	
**DIAMOnD**							0,11	
**MaxLink**								

	TOPAS(1)	TOPAS(2)	TOPAS(3)	SCA	ROBUST	DIAMOnD	MaxLink	
**TOPAS(1)**		0,42	0,40	**0,46**	0,19	0,01	0,05	**Drug targets**
**TOPAS(2)**			0,75	0,32	0,27	0,02	0,05	
**TOPAS(3)**				0,30	0,28	0,02	0,05	
**SCA**					0,24	0,02	0,07	
**ROBUST**						0,05	0,12	
**DIAMOnD**							0,11	
**MaxLink**								

	TOPAS(1)	TOPAS(2)	TOPAS(3)	SCA	ROBUST	DIAMOnD	MaxLink	
**TOPAS(1)**		0,66	0,65	**0,43**	0,27	0,01	0,01	**KEGG diseases**
**TOPAS(2)**			0,91	0,36	0,30	0,01	0,01	
**TOPAS(3)**				0,36	0,30	0,01	0,01	
**SCA**					0,35	0,01	0,02	
**ROBUST**						0,02	0,04	
**DIAMOnD**							0,16	
**MaxLink**								

Szymkiewicz–Simpson coefficient

	TOPAS(1)	TOPAS(2)	TOPAS(3)	SCA	ROBUST	DIAMOnD	MaxLink
**TOPAS(1)**		0,70	0,69	0,70	**0,93**	0,35	0,44
**TOPAS(2)**			0,84	0,48	0,86	0,24	0,29
**TOPAS(3)**				0,47	0,84	0,21	0,26
**SCA**					0,76	0,26	0,38
**ROBUST**						0,18	0,35
**DIAMOnD**							0,48
**MaxLink**							

70 diseases

	TOPAS(1)	TOPAS(2)	TOPAS(3)	SCA	ROBUST	DIAMOnD	MaxLink
**TOPAS(1)**		0,70	0,71	0,75	0,88	0,49	0,58
**TOPAS(2)**			0,87	0,53	**0,89**	0,38	0,47
**TOPAS(3)**				0,51	0,87	0,37	0,45
**SCA**					0,78	0,40	0,54
**ROBUST**						0,31	0,55
**DIAMOnD**							0,11
**MaxLink**							

Drug targets

	TOPAS(1)	TOPAS(2)	TOPAS(3)	SCA	ROBUST	DIAMOnD	MaxLink
**TOPAS(1)**		0,85	0,85	0,70	**0,87**	0,26	0,70
**TOPAS(2)**			0,96	0,58	0,83	0,24	0,60
**TOPAS(3)**				0,57	0,83	0,24	0,59
**SCA**					0,84	0,18	0,70
**ROBUST**						0,14	0,54
**DIAMOnD**							0,66
**MaxLink**							

KEGG diseases

Supplementary Table 8. Consistency between networks as averaged Jaccard-Index (JI) and Szymkiewicz–Simpson coefficient (SS) throughout networks. The maximum overlap is highlighted in bold.

		TOPAS(1)	TOPAS(2)	TOPAS(3)	SCA	ROBUST	DIAMOnD	MaxLink
**JI**	**70 diseases**	0,41	0,44	**0,45**	0,36	0,23	0,13	0,11
	**Drug targets**	0,41	**0,45**	**0,45**	0,38	0,23	0,11	0,09
	**KEGG diseases**	**0,78**	**0,78**	**0,78**	0,74	0,59	0,27	0,20
**SS**	**70 diseases**	**0,67**	0,66	0,66	0,63	0,43	0,21	0,44
	**Drug targets**	**0,70**	0,66	0,66	0,67	0,48	0,18	0,34
	**KEGG diseases**	**0,90**	0,89	0,89	0,87	0,80	0,42	0,54

Supplementary Figures

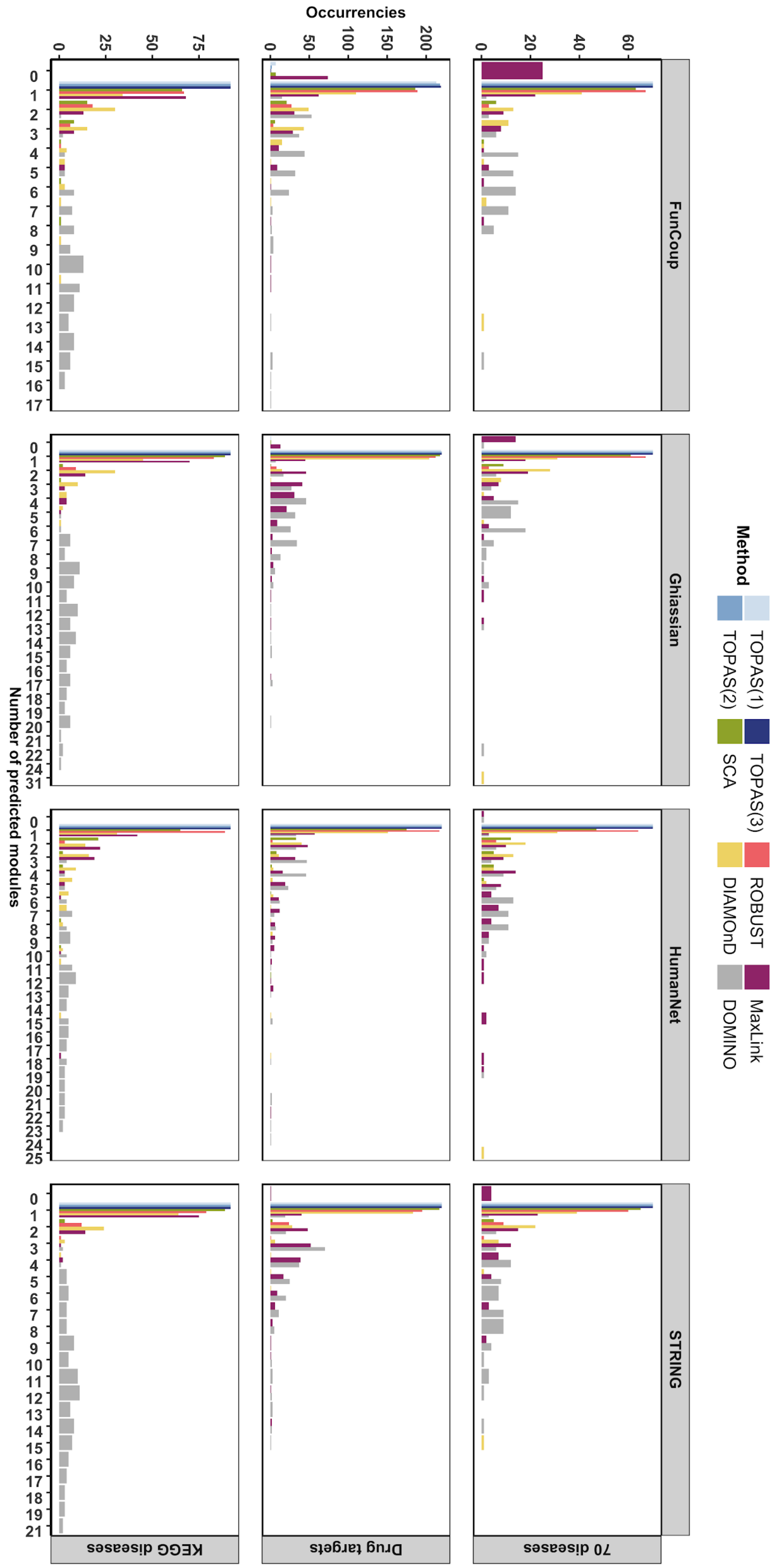

Supplementary Figure 1. Number of predicted modules per method.

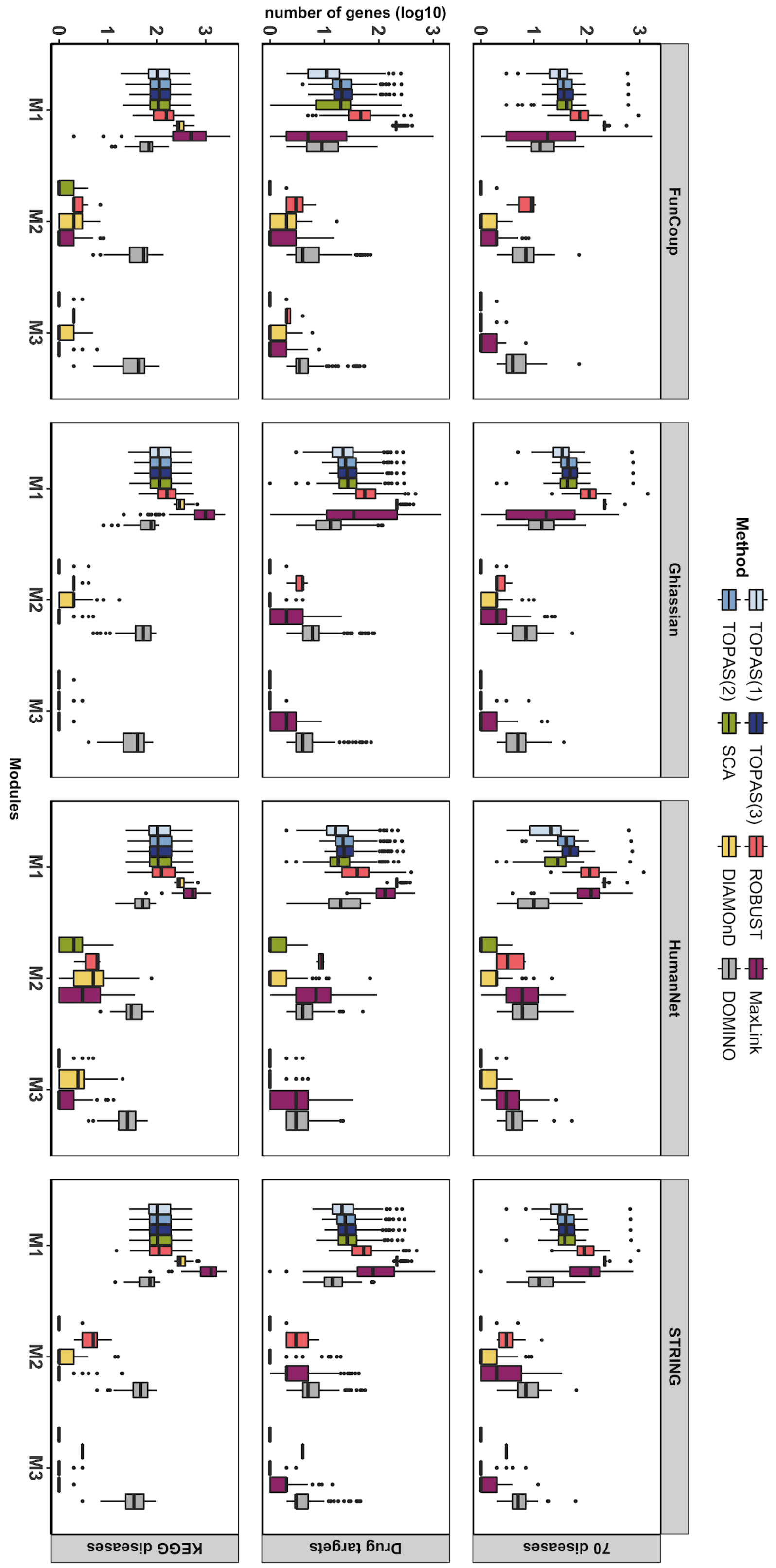

Supplementary Figure 2. TOP 3 largest predicted seed modules.

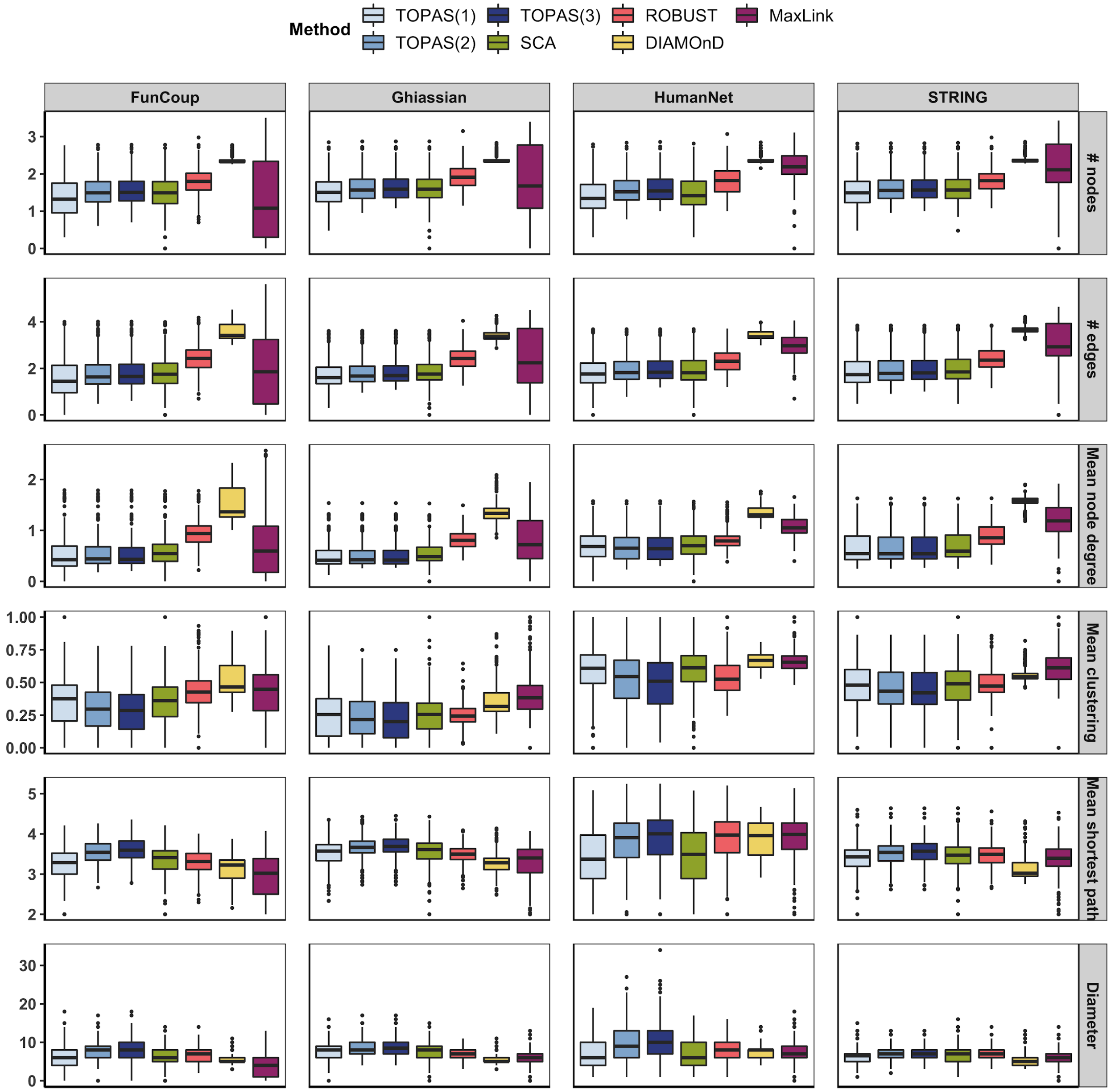

Supplementary Figure 3. Predicted Modules properties overview. Number of links, number of nodes and mean degree nodes are shown as log10-transformed values.

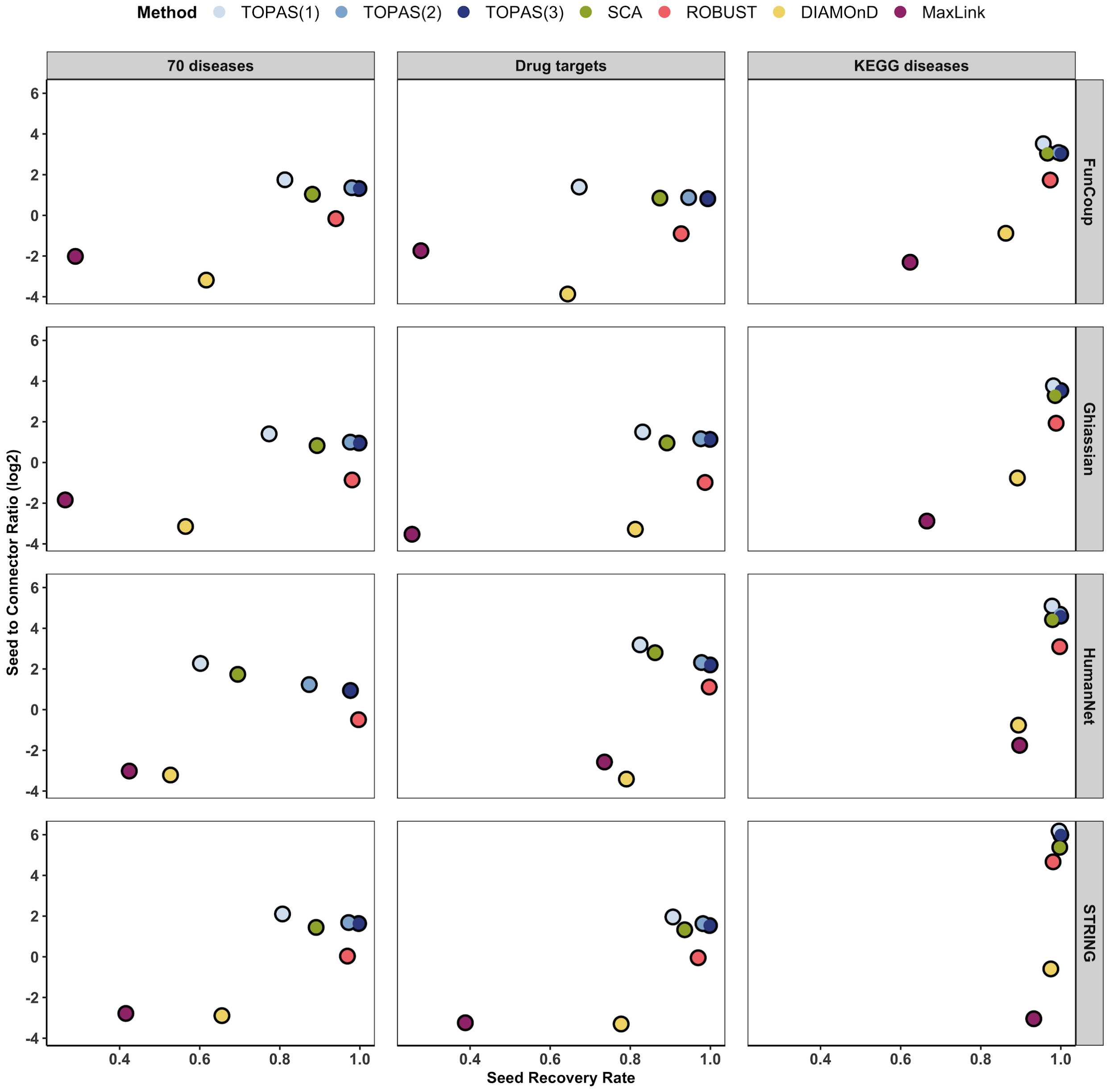

Supplementary Figure 4. Average Seed to Connector Ratio vs Seed Recovery Rate.

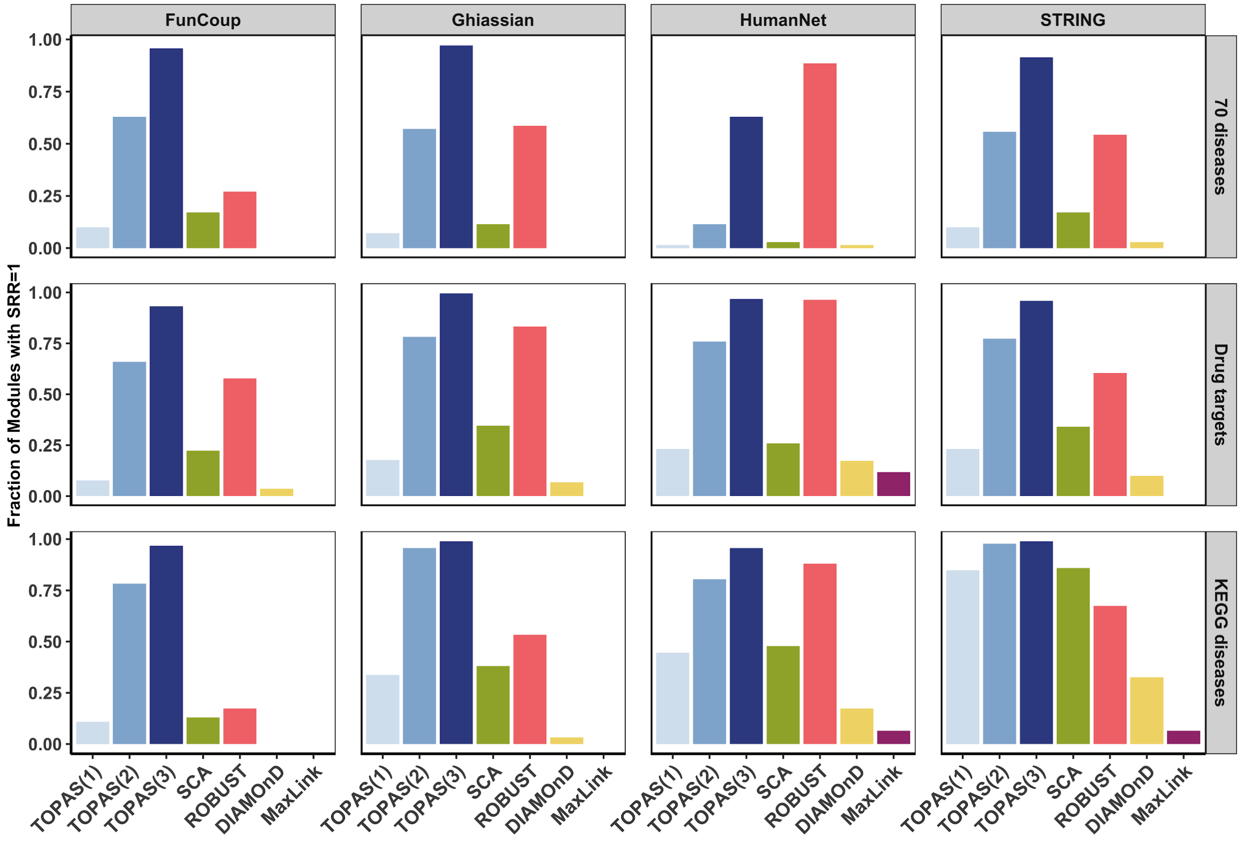

Supplementary Figure 5. Ratio of modules with full Seed Recovery Rate.

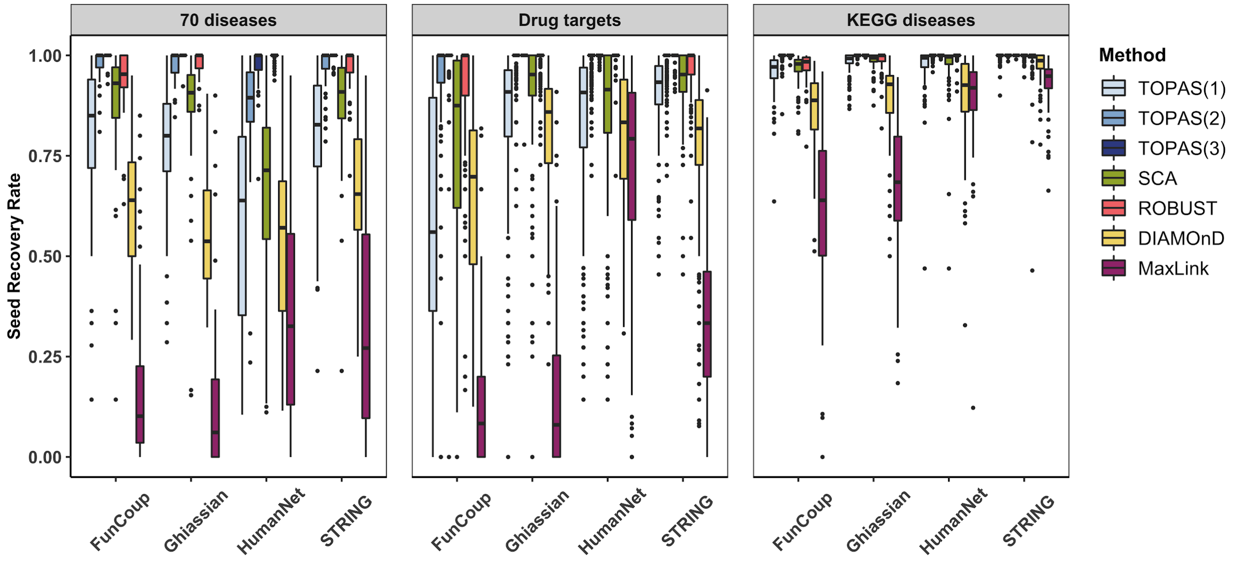

Supplementary Figure 6. All Seed Recovery Rates. The distribution of SRR when using the four different networks.

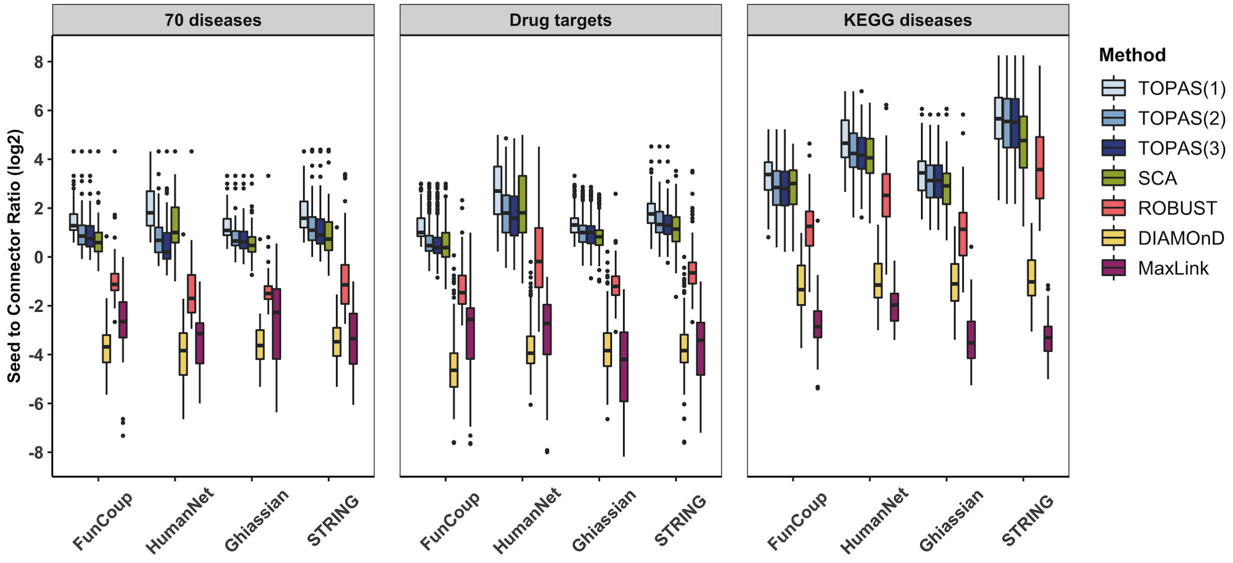

Supplementary Figure 7. Seeds/Connectors Ratio. The complete distribution of SCR.

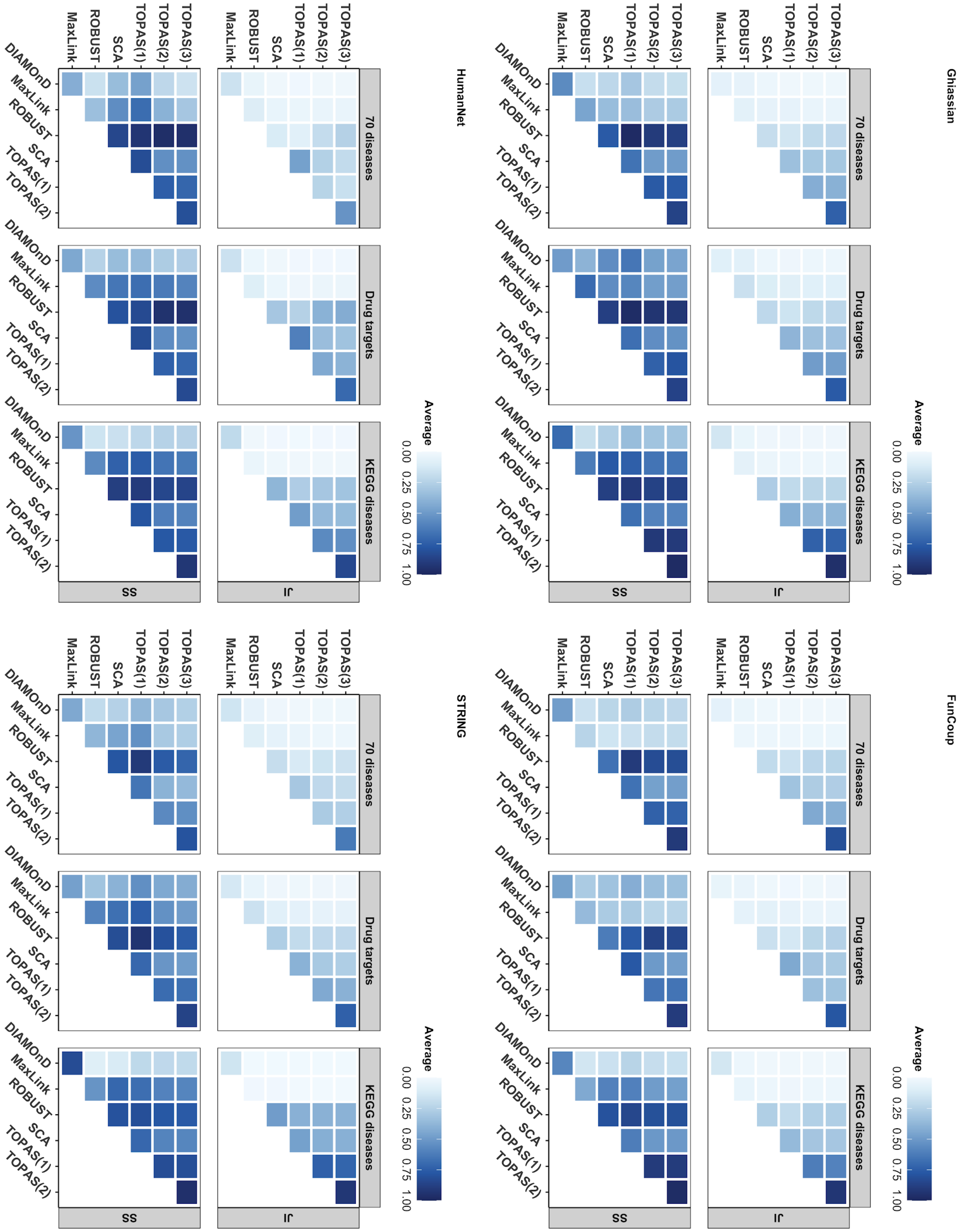

Supplementary Figure 8. Consistency between methods. We measured the overlap of connector genes in the predicted modules between every pair of methods by using the same network. Overall, DIAMOnD stands out from the rest by inferring more dissimilar modules.

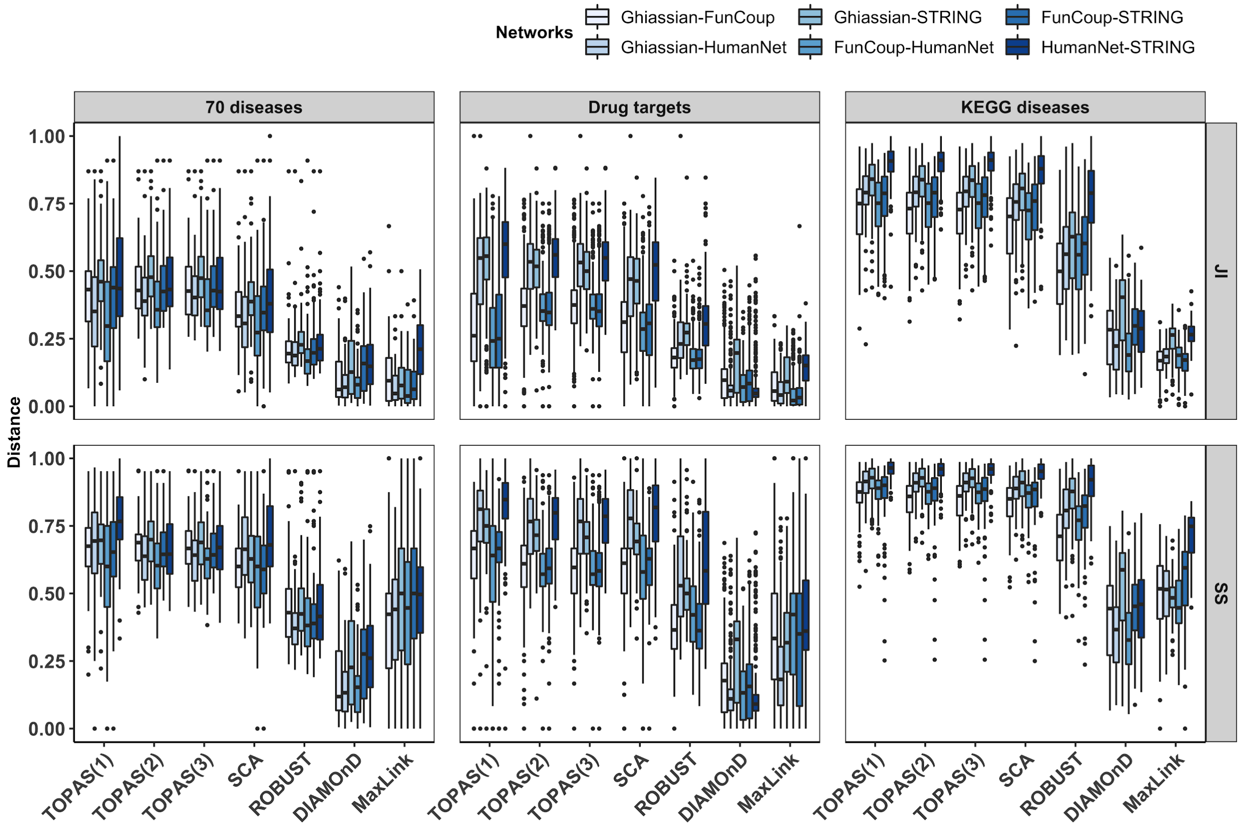

Supplementary Figure 9. Consistency between networks. For each method, we measured the overlap of genes in the predicted modules between every pair of networks.

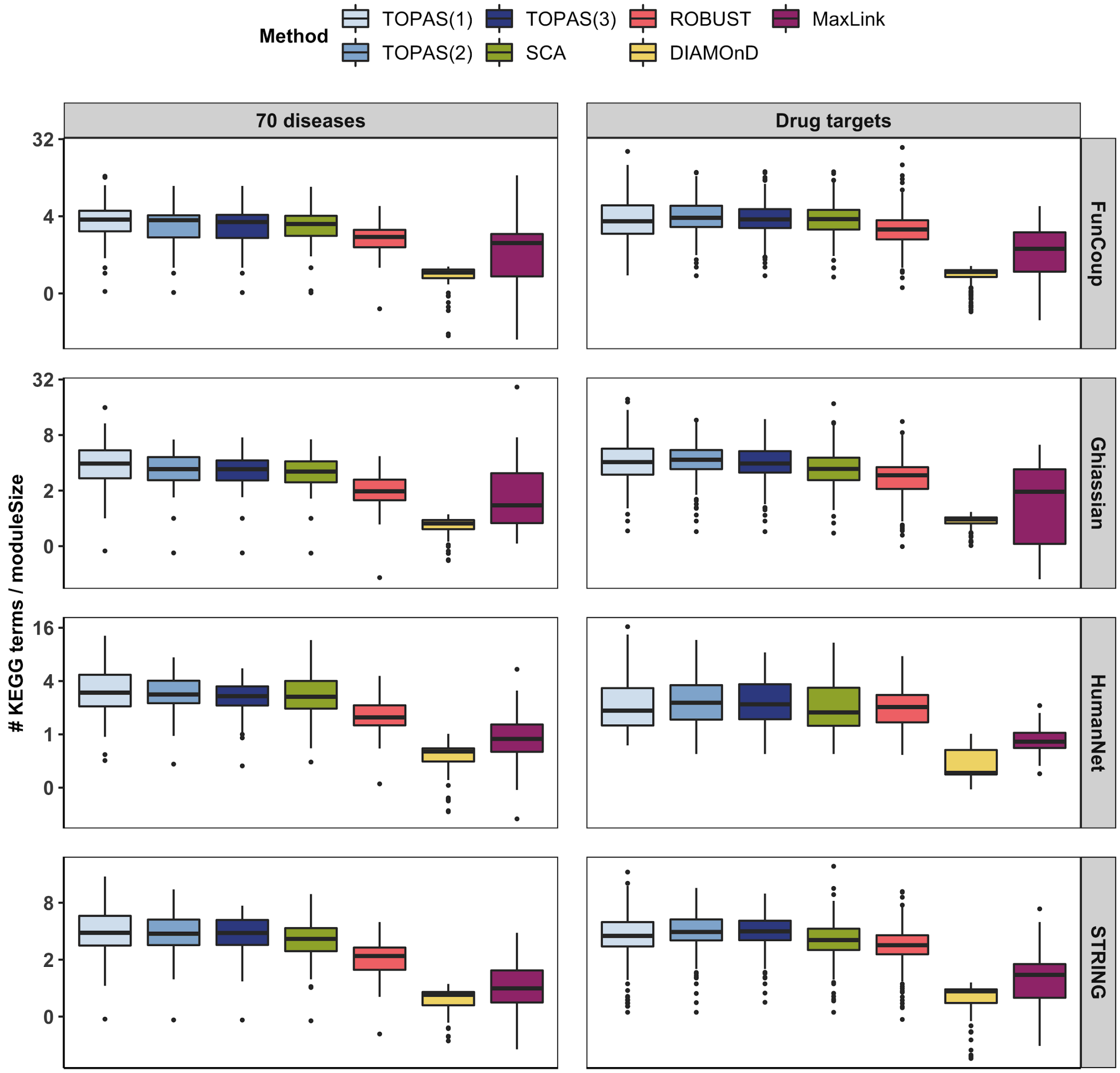

Supplementary Figure 10. KEGG pathway terms diversity.
